# Emergence of unusual coexistence states in cyclic game systems

**DOI:** 10.1038/s41598-017-07911-4

**Published:** 2017-08-07

**Authors:** Junpyo Park, Younghae Do, Bongsoo Jang, Ying-Cheng Lai

**Affiliations:** 1 Department of Mathematical Sciences, Ulsan National Institute of Science and Technology, Ulsan, 44919 Republic of Korea; 2 Department of Mathematics, KNU-Center for Nonlinear Dynamics, Kyungpook National University, Daegu, 41566 Republic of Korea; 3 School of Electrical, Computer, and Energy Engineering, Arizona State University, Tempe, Arizona 85287 USA

## Abstract

Evolutionary games of cyclic competitions have been extensively studied to gain insights into one of the most fundamental phenomena in nature: biodiversity that seems to be excluded by the principle of natural selection. The Rock-Paper-Scissors (RPS) game of three species and its extensions [e.g., the Rock-Paper-Scissors-Lizard-Spock (RPSLS) game] are paradigmatic models in this field. In all previous studies, the intrinsic symmetry associated with cyclic competitions imposes a limitation on the resulting coexistence states, leading to only selective types of such states. We investigate the effect of nonuniform intraspecific competitions on coexistence and find that a wider spectrum of coexistence states can emerge and persist. This surprising finding is substantiated using three classes of cyclic game models through stability analysis, Monte Carlo simulations and continuous spatiotemporal dynamical evolution from partial differential equations. Our finding indicates that intraspecific competitions or alternative symmetry-breaking mechanisms can promote biodiversity to a broader extent than previously thought.

## Introduction

Fundamental to species coexistence and biodiversity are competitions. In ecosystems there are two types of competitions: interspecific (competitions among individuals from different species) and intraspecific (competitions among individuals in the same species), where both types can either promote or hinder species coexistence^1, 2^. The purpose of this paper is to demonstrate, through a systematic study of several models of cyclic evolutionary game, that intraspecific competitions can induce unusual states of coexistence that have not been reported previously. Intraspecific competitions may thus be more fundamental to biodiversity than previously thought.

A natural and typical mechanism for interspecific competitions is predator-prey interaction, while intraspecific competitions occur because individuals in the same species compete for essential life-sustaining resources such as food, water, light, and opposite sex. A well known type of intraspecific competitions is cannibalism or intraspecific predation^3–10^, which can occur with high likelihood especially when there is lack of sufficient resources. Such competitions can also occur when individuals fight each other for mating opportunities, which were observed for side-blotched lizards in California^11^. In the past decade there were studies of the effect of intraspecific competitions on biodiversity^12–16^, with results such as the experimental finding that the competitions tend to drive disruptive selection^12^, enhanced host survival through intraspecific competition between co-infecting parasite strains^13^, and directional selection of certain fish species^15, 16^.

To understand coexistence and biodiversity, the approach of mathematical modeling has proven to be useful, providing fundamental insights into the various mechanisms underlying species coexistence at both the macroscopic, population^17–19^ and the microscopic, individual competition levels^20–23^. Historically, the theoretical approach began with mathematically modeling growth and competitions through dynamical equations at the population level^17–19^. In the past fifteen years or so, microscopic models at the level of individual interactions were extensively studied based on the mathematical paradigm of evolutionary games^24–53^. A milestone result^22, 26^ is the elucidation of the role of species mobility in coexistence, which traditionally had been regarded as detrimental to coexistence. In particular, utilizing the framework of three cyclic competing species, the rock-paper-scissors (RPS) model, the authors^22, 26^ demonstrated robust coexistence in the weak mobility regime, providing a resolution to the paradox that macroscopic models exclude coexistence of mobile species but, in realistic ecological processes ranging from bacteria run and tumble to animal migration, coexistence is ubiquitous. The basic dynamical structure supporting the coexistence of mobile species was identified to be spiral wave patterns that emerge and evolve with time in the physical space^22^, which are robust against noise^26^. Other issues that have been investigated include the stability of spatial patterns^24, 29, 39, 52, 54–57^, the role of conservation laws^27, 44^, pairwise and group-level interactions^53^, basins of the coexistence states^30, 31^, and the effects of a wide array of behaviors/quantities on coexistence such as long range migration^38, 45^, uniform intraspecific competition^32^, local habitat suitability^46^, multi-strategy competition^49^, inhomogeneous reaction rates^28, 33, 40, 47, 48^, epidemic spreading^34^, and spatial extent and population size^25, 36, 41^. While most of these works were for three cyclic competing species, there were studies extending the model to arbitrary number of species^42, 43, 53^ and addressing the role of competition at the mesoscopic (i.e., group) level in coexistence^52^. Here extinction means no coexistence and only one surviving species in the system.

In previous works, a well established result is that only a certain type of coexistence states can exist. For example, in a system of three cyclically competing species (RPS game), the only coexistence state is one that involves all three species: it is not possible for a state of two coexisting species^22, 26^ to be stable. Likewise, in a cyclic system of five species, there are two distinct coexistence states with either three or five species - there cannot be coexistence states with two or four species^52^. The reason for the selective coexistence states lies in the intrinsic symmetry of the system, as the competing species are at the same footing. Intuitively, symmetry breaking can possibly lead to more diverse coexistence states. The purpose of this paper is to demonstrate and establish that realistic intraspecific competitions that generically break the intrinsic symmetry of cyclic competitions, can lead to *all possible coexisting states*. In particular, in the real world the degree of intraspecific competition in general depends on the particular species. To describe symmetry-breaking in a concrete way, we use a parameter, e.g., the rate of intraspecific competition. The rate can then be nonuniform for different species, which can have a significant effect on the game dynamics^33, 58–61^. At the microscopic level, the species are thus no longer on the equal footing - effectively introducing symmetry breaking into the system. As a result, coexisting states without a global symmetry at the macroscopic level can arise. We establish this striking result through extensive computations and mathematical analyses of cyclically competing systems with different number of species.

## Results

### Models and mathematical representations

**Figure 1:**
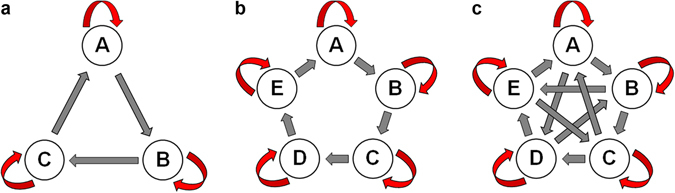
Cyclic games with intraspecific competitions. (**a**) Rock-paper-scissors (RPS) game of three species, (**b**) Extended Rock-Paper-Scissors (ERPS) game of five species, and (**c**) Rock-Paper-Scissors-Lizard-Spock (RPSLS) game of five species. Straight and looped arrows indicate interspecific and intraspecific competitions, respectively.

To investigate the dynamical evolutions of cyclically competing species in the presence of intraspecific competitions, we study three game systems: the classic RPS model, the extended RPS (ERPS) model of five species, and the rock-paper-scissors-lizard-spock (RPSLS) model. The dynamical interacting rules of the three models are shown in Fig. 1. At the microscopic level, each model can be described by evolutionary dynamics on a lattice system, while at the macroscopic level the model can be approximated by a set of ordinary differential equations (ODEs). In addition, partial differential equations (PDEs) can be used to study the spatiotemporal evolution of the population densities.

For convenience, we employ a square lattice with periodic boundary conditions to host a cyclic game system at the microscopic level, where an individual occupies a lattice site. Given the interspecific competition rate *σ*, species reproduction rate *μ*, intraspecific competition rate *p*, and movement rate *ε*, interactions among the individuals can be described by

1 $$XY\mathop{\to }\limits^{\sigma }X\varnothing ,\quad X\varnothing \mathop{\to }\limits^{\mu }XX,\quad XX\mathop{\to }\limits^{p}X\varnothing ,\quad XZ\mathop{\to }\limits^{\varepsilon }ZX,$$

where *X* and *Y* represent two cyclically interacting species, $\varnothing$ denotes an empty site, and *Z* stands for either an individual from the same species or an empty site, and the quantity *ε* is defined to be *ε* ≡ 2*MN* with *M* being the individual mobility parameter and *N* being the total number of individuals. In the evolutionary game system, each site can be occupied by an individual from one of the species or left empty. At each time step, a randomly selected individual can compete, reproduce, or move into one of its nearest neighbors at random, provided that the corresponding interaction rule as specified in Eq. (1) is satisfied.

Under the mean field approximation where the population size is large: *N* → ∞, the system can be described by the rate equations governing the time evolution of the species densities (ODEs). Let *a*(*t*), *b*(*t*), and *c*(*t*) be the densities of the three species *A*, *B*, and *C* at time *t*, respectively. The RPS game model can be described by

2 $$\begin{array}{rcl}\frac{da}{dt} & = & a\,[\mu \mathrm{(1}-\rho )-\sigma c-\frac{{p}_{a}}{2}a],\\ \frac{db}{dt} & = & b\,[\mu \mathrm{(1}-\rho )-\sigma a-\frac{{p}_{b}}{2}b],\\ \frac{dc}{dt} & = & c\,[\mu \mathrm{(1}-\rho )-\sigma b-\frac{{p}_{c}}{2}c],\end{array}$$

where *ρ*(*t*) ≡ *a*(*t*) + *b*(*t*) + *c*(*t*) is the total density of the three species. In each equation, the first and second terms describe reproduction of a species at rate *μ* and a decrease in the species density by invasion at rate *σ*, respectively. The third term represents the decrease in the density due to intraspecific competition of rates *p*_*a*_, *p*_*b*_, or *p*_*c*_ for species *A*, *B* and *C*, respectively, where the factor 1/2 associated with the rate accounts for the two-way interactions between two individuals in the same species. (ODE models for ERPS and RPSLS games are provided in Supplementary Information).

The ODE model ignores the effects of the spatial domain in which the interactions occur. To take into account the spatial dimension, a PDE model can be derived. In particular, consider a square domain of linear size *L* with periodic boundary conditions, where *L* ^2^ = *N*. We normalize the domain to the unit square so that the distance between two nearest neighbors is $\delta x=1/\sqrt{N}=(1/L)$. The densities of subpopulations *A*, *B*, and *C* at time *t* and site **x** = (*x*_1_, *x*_2_) are denoted as *a*(**x**, *t*), *b*(**x**, *t*), and *c*(**x**, *t*), respectively. For interspecific and intraspecific competitions as well as reproduction, the dynamical equations for these quantities only involve neighbors located at **x** ± *δx* · **e**_*i*_, where {**e**_*i*_}_*i*=1,2_ are the base vectors of the lattice. For the RPS game, we obtain the following evolutionary equations:

3 $$\begin{array}{l}\tfrac{\partial a({\bf{x}},t)}{\partial t}=M\,{\rm{\Delta }}a\,({\bf{x}},t)+\mu a\,({\bf{x}},t)\,\mathrm{[1}-\rho ({\bf{x}},t)]-\sigma a\,({\bf{x}},t)\,c\,({\bf{x}},t)-\tfrac{{p}_{a}}{2}a\,({\bf{x}},t)\,a\,({\bf{x}},t),\\ \tfrac{\partial b({\bf{x}},t)}{\partial t}=M\,{\rm{\Delta }}b\,({\bf{x}},t)+\mu b\,({\bf{x}},t)\,\mathrm{[1}-\rho ({\bf{x}},t)]-\sigma b\,({\bf{x}},t)\,a\,({\bf{x}},t)-\tfrac{{p}_{b}}{2}b\,({\bf{x}},t)\,b\,({\bf{x}},t),\\ \tfrac{\partial c({\bf{x}},t)}{\partial t}=M\,{\rm{\Delta }}c\,({\bf{x}},t)+\mu c\,({\bf{x}},t)\,\mathrm{[1}-\rho ({\bf{x}},t)]-\sigma b\,({\bf{x}},t)\,c\,({\bf{x}},t)-\tfrac{{p}_{c}}{2}c\,({\bf{x}},t)\,c\,({\bf{x}},t\mathrm{)}.\end{array}$$

where $M=\frac{\varepsilon }{2}{(1/\sqrt{N})}^{2}$. (The corresponding PDEs for ERPS and RPSLS games are provided in Supplementary Information).

To be concrete, in this paper we fix the rates of interspecific competition and reproduction to be *σ* = 1 and *μ* = 1, respectively.

### Coexistence states in the RPS system

**Figure 2:**
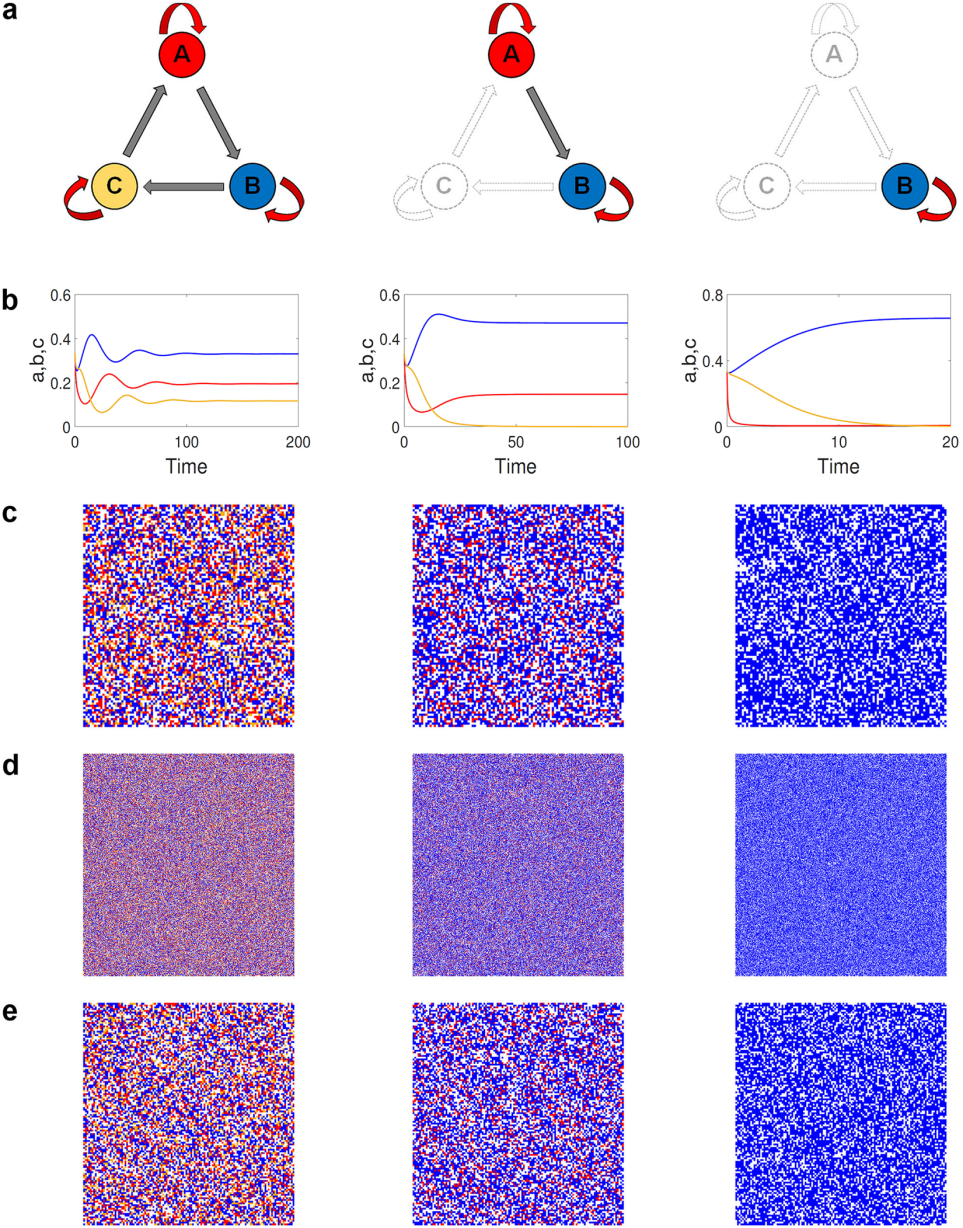
All possible coexistence states in RPS game. Red, blue and yellow colors indicate different species and blank denotes empty sites. (**a**) Three different types of surviving species. The left column corresponds to *p*_*a*_ = 2.5, where (**b**) shows the density evolution from the ODE model, (**c**) and (**d**) present typical snapshots obtained from Monte Carlo simulations of lattice size *N* = 100 × 100 and 500 × 500, respectively, and (**e**) is a snapshot obtained from the PDE model. The middle and right columns are for *p*_*a*_ = 5.2 and *p*_*a*_ = 100, respectively. Other parameters are *p*_*b*_ = 1.0 and *p*_*c*_ = 0.5.

**Figure 3:**
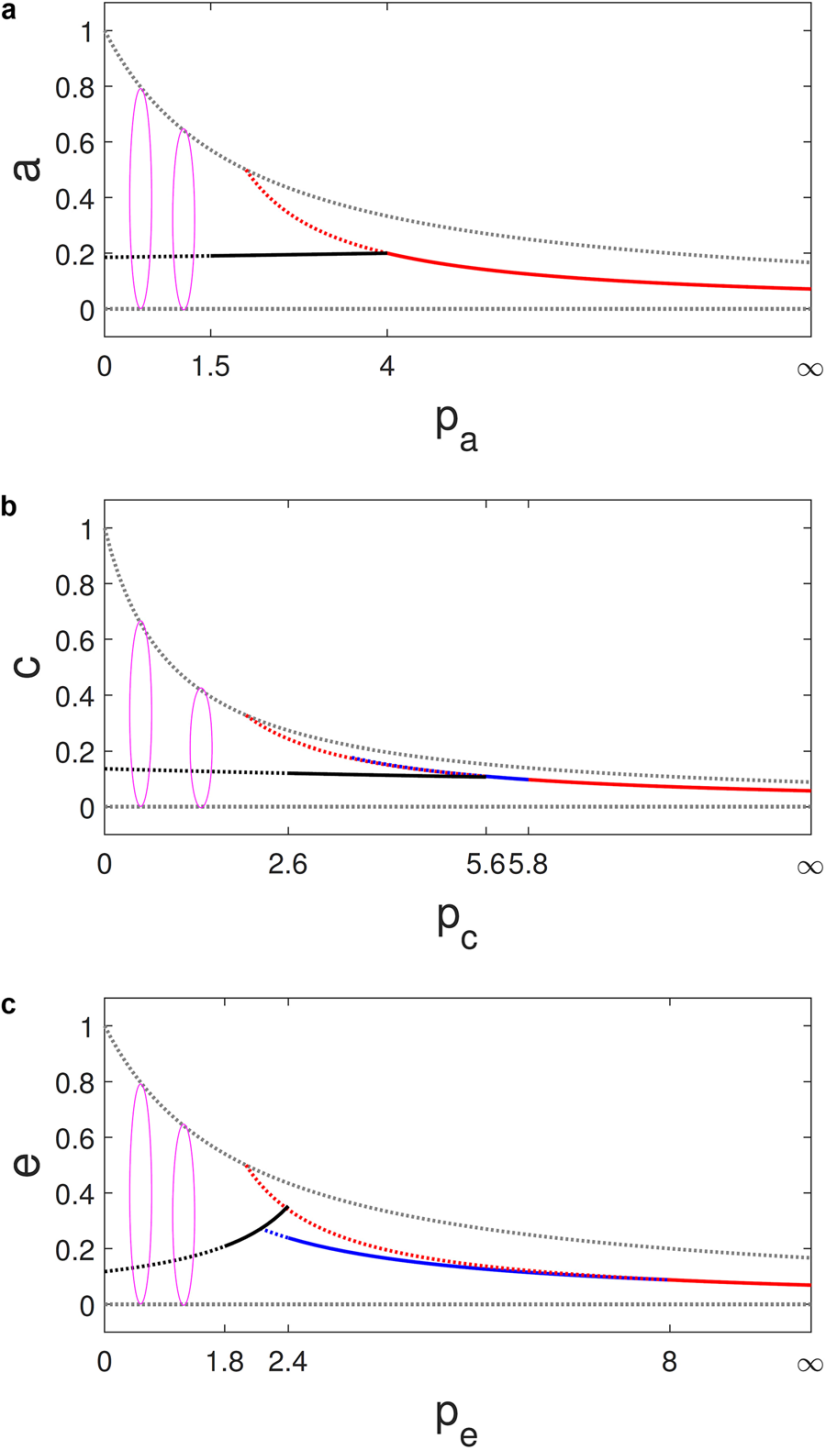
Bifurcation with intraspecific parameter. Solid lines and ellipses indicate stable fixed points and heteroclinic cycles, respectively, and dots are for unstable fixed points. Different fixed points are distinguished by colors. (**a**) For the RPS game, the survival states of three and two species (black and red lines), respectively. (**b**) For the ERPS game, black, blue and red lines indicate the survival states of five, four, and three species, respectively. (**c**) For the RPSLS game, black, blue and red lines specify the survival states of five, four and three species, respectively.

Figure 2 illustrates the possible coexistence states in the RPS system, where the middle column represents a state in which two species coexist - a previously unknown coexistence state. Specifically, a stability analysis of the corresponding ODE system with respect to systematic changes in a intraspecific competition parameter, say *p*_*a*_, with fixed *p*_*b*_ = 1 and *p*_*c*_ = 0.5, reveals the following phenomena. Firstly, for weak intraspecific competition, i.e., 0 < *p*_*a*_ ≤ 1.5, coexistence is physically not possible due to the existence of a heteroclinic cycle, where only one species can survive. Secondly, for moderate intraspecific competition, i.e., 1.5 < *p*_*a*_ ≤ 4, for each species reproduction and death are counter-balanced, so all three species can coexist, as indicated by the left column in Fig. 2. Thirdly, for stronger intraspecific competition, i.e., *p*_*a*_ > 4, the new coexistence state of two species emerges, as indicated by the middle column in Fig. 2. The striking consequence is that, in this case, the nature of the interaction between the two surviving species has become the predator-prey type as a result of the balance between prey’s reproduction and predator’s death from competitions among its own individuals. In the limit of infinitely strong intraspecific competition, i.e., *p*_*a*_ → ∞, the predator becomes extinct, leaving the prey as the only surviving species, as shown in the right column of Fig. 2. Figure 3(a) presents a bifurcation diagram of these behaviors (see Methods for more details).

The spatial patterns associated with distinct coexisting states can be revealed by numerical solutions of the lattice and PDE models, Eqs (1) and (3), respectively. Figure 2(c–e) show, for *M* = 10^−3^, nine representative snapshots of the spatial patterns in the long time regime for different values of *p*_*a*_, where the three species are denoted by red, blue, and yellow, respectively. Specifically, the three columns (from left to right) are associated with *p*_*a*_ = 2.5, 5.2 and 100, respectively, the two top rows [Fig. 2(c,d)] are from lattice models of size *L* = 100 × 100 and 500 × 500, respectively, and the bottom row is from the PDE model. We see that, all possible coexistence states can occur and, as the intraspecific competition parameter is increased, physical coexistence states for which the number of survival species is in the order 1 → 3 → 2 → 1 emerge, which is consistent with the results from the bifurcation analysis of the ODE model. Further, we find that the thresholds between different stable phases from lattice simulations are consistent with those from the bifurcation analysis of the ODE model, as supported by a calculation of the survival probability *P*_*surv*_ from 100 sampled parameter values (see Fig. S9 in Supplementary Information). The middle column shows the patterns of the coexistence state of two species in the spatial domain, which has not been observed previously in the cyclic game of three species. Previous studies also revealed that the coexistence state of three species is supported by spiral waves in the domain^22, 26^. In general, spiral waves can be stable, unstable or convectively unstable even in the absence of intraspecific competitions^54–56^. However, with intraspecific competition induced symmetry breaking at the microscopic level, various coexistence states can arise but no spiral wave patterns can form.

### Coexistence states in the ERPS system

There are five competing species in the ERPS system, as shown in Fig. 1(b). To be concrete, we consider a fixed set of parameter values: *p*_*a*_ = 1.9, *p*_*b*_ = 2, *p*_*d*_ = 1.3, and *p*_*e*_ = 0.7. A bifurcation analysis of the underlying ODE model (Supplementary Information) leads to the following results on the role of intraspecific competition in coexistence. Firstly, for weak intraspecific competition, i.e., 0 ≤ *p*_*c*_ < 2.6, mathematically all species can coexist but small perturbations can lead to extinction, physically excluding coexistence. Secondly, for a moderate level of intraspecific competition, i.e., 2.6 ≤ *p*_*c*_ < 5.6, all species can physically coexist. Thirdly, in the regime of strong intraspecific competition, i.e., 5.6 < *p*_*c*_ < 5.8, coexistence states of four species can emerge, breaking the cyclic symmetry. Fourthly, for stronger intraspecific competition, i.e., *p*_*c*_ ≥ 5.8, three species can coexist, exhibiting a predator-prey relation. For *p*_*c*_ → ∞, the predator populations diminish and only two species can coexist. Figure 3(b) presents a bifurcation diagram of these behaviors.

**Figure 4:**
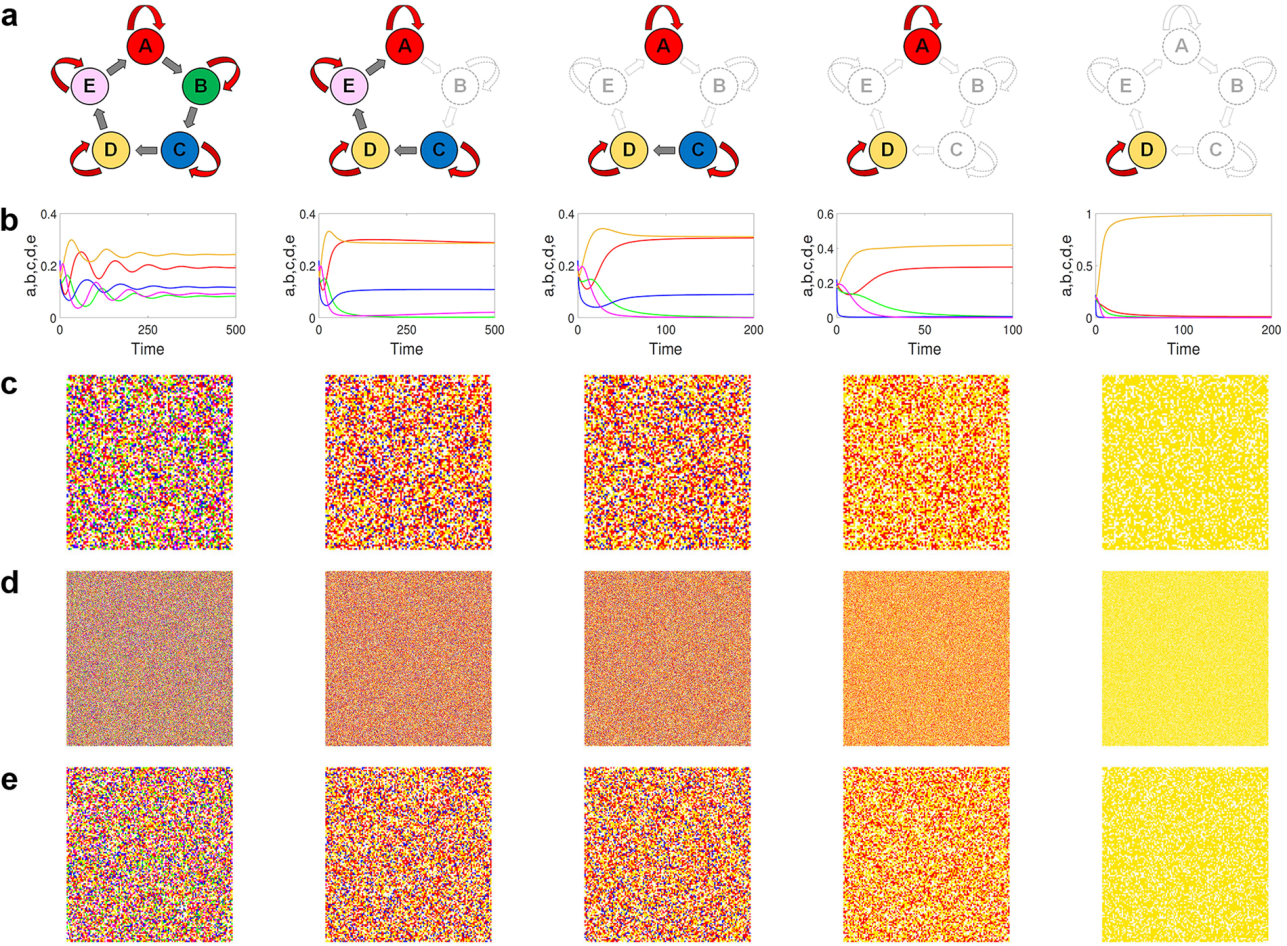
Coexistence states in ERPS game. (**a**) Five different types of surviving species, where each color denotes a different species (blank for empty site). The four columns from left correspond to four different values of the intraspecific competition rate of one species: *p*_*c*_ = 3.3, 5.65, 6.5 and 100, respectively, with fixed parameters *p*_*a*_ = 1.9, *p*_*b*_ = 2.0, *p*_*d*_ = 1.3, and *p*_*e*_ = 0.7. For example, in the left most column, (**b**) is the density evolution from the ODE model, (**c**,**d**) are typical snapshots obtained from Monte Carlo simulations of lattice size *N* = 100 × 100 and 500 × 500, respectively, and (**e**) is a snapshot obtained from the PDE model. The right most column is for *p*_*a*_ = 1.9, *p*_*b*_ = 2.0, *p*_*c*_ = 100, *p*_*d*_ = 0.01, and *p*_*e*_ = 0.7.

The emergence of all possible coexistence states, especially the unusual states of three and four coexisting species, can be substantiated by resorting to the lattice and PDE models (see Supplementary Information for the PDE model of the ERPS system). To be concrete, we set the simulation parameters to be *p*_*a*_ = 1.9, *p*_*b*_ = 2, *p*_*d*_ = 1.3, *p*_*e*_ = 0.7, and *M* = 10^−3^. Figure 4(c–e) show fifteen snapshots of the spatial patterns in the long time regime from the lattice and PDE models for a number of different values of *p*_*c*_, where the five species are denoted by red, blue, green, yellow and pink, respectively. The panels are organized into rows and columns, where columns 1–4 (from left to right) are associated with *p*_*c*_ = 3.3, 5.65, 6.5 and 100, and column 5 is for *p*_*c*_ = 100 but with a different parameter setting (*p*_*a*_ = 1.9, *p*_*b*_ = 2, *p*_*d*_ = 0.01, and *p*_*e*_ = 0.7). The two top rows [Fig. 4(c,d)] are the results from lattices of size *L* = 100 × 100, 500 × 500, respectively, and the bottom row represents the results from the PDE model. These results are consistent with those from the stability analysis of the corresponding ODE system in that coexistence states of all possible numbers of species can occur. The most striking phenomenon is the coexistence of four and three species (corresponding to the second and third columns, respectively), which have not been reported previously for the ERPS system. Similar to the RPS system, with intraspecific competitions the coexistence states are not supported by spiral wave patterns in the spatial domain.

### Coexistence states in the RPSLS system

**Figure 5:**
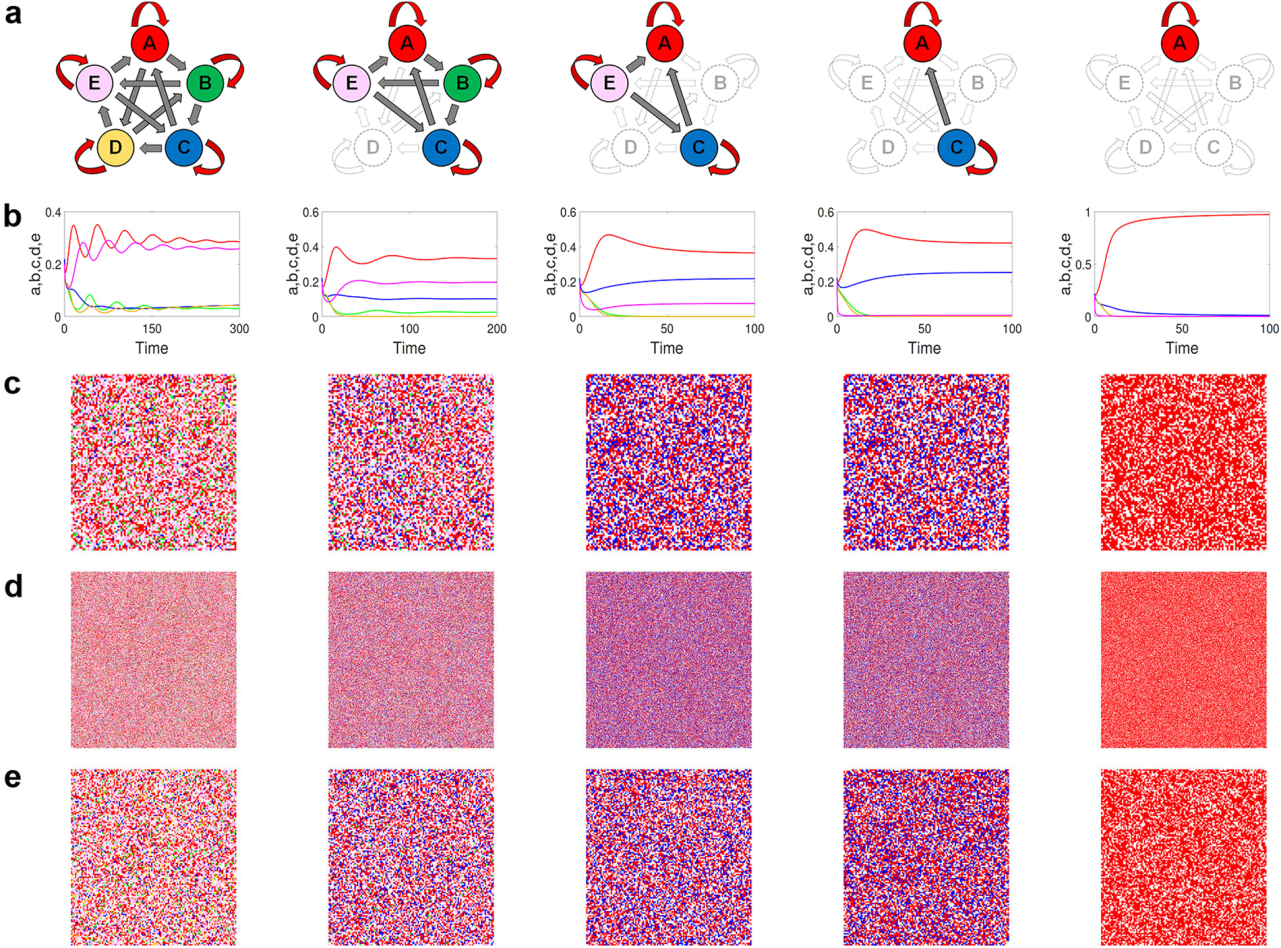
Coexistence states in RPSLS game. (**a**) Five different types of surviving species, where each color denotes a different species (blank for empty site). The four columns from left correspond to four different values of the intraspecific competition rate of one species: *p*_*e*_ = 2.2, 3.3, 9.3 and 100, respectively, with fixed parameters *p*_*a*_ = 0.3, *p*_*b*_ = 1.1, *p*_*c*_ = 2.5 and *p*_*d*_ = 0.7. For example, in the left most column, (**b**) is the density evolution from the ODE model, (**c**,**d**) are typical snapshots obtained from Monte Carlo simulations of lattice size *N* = 100 × 100 and 500 × 500, respectively, and (**e**) is a snapshot obtained from the PDE model. The right most column is for *p*_*a*_ = 0.01, *p*_*b*_ = 1.1, *p*_*c*_ = 2.5, *p*_*d*_ = 0.7, and *p*_*e*_ = 100.

For the five-species RPSLS system, for a representative set of fixed parameter values, e.g., *p*_*a*_ = 0.3, *p*_*b*_ = 1.1, *p*_*c*_ = 2.5, and *p*_*d*_ = 0.7, but varying *p*_*e*_, a stability analysis reveals the following phenomena. Firstly, in the regime of weak intraspecific competition, i.e., 0 ≤ *p*_*e*_ < 1.8, coexistence is physically not possible. Secondly, for 1.8 ≤ *p*_*e*_ < 2.4, coexistence of all five species is physically realizable and robust. Thirdly, for 2.4 ≤ *p*_*e*_ < 8, coexistence of five species is no longer possible. Instead, coexistence states of four species can emerge, breaking the cyclic symmetry. Fourthly, for *p*_*e*_ ≥ 8, only three species, which do not exhibit a sub-cyclic structure, can coexist and, for *p*_*e*_ → ∞, two of the three species can survive in a predator-prey relation. Figure 3(c) presents a bifurcation diagram of these behaviors. Detailed simulations from the lattice and PDE (Supplementary Information) models give consistent results. For example, for *p*_*a*_ = 0.3, *p*_*b*_ = 1.1, *p*_*c*_ = 2.5, and *p*_*d*_ = 0.7, various coexistence states can emerge for different values of *p*_*e*_, as shown by the spatial patterns in Fig. 5(c–e). The results shown in the second and fourth columns, which indicate the coexistence states of four and two species, are surprising as such states have not been uncovered previously in the study of RPSLS system^52^. We also find that, associated with the coexistence of three species [c.f., Fig. 5(a)], there is absence of any cyclic interaction structure among the three survived species. This is in fact a *non*-*sub*-*cyclic interacting structure* which, to our knowledge, has not been reported previously in the studies of interspecific interaction models^52, 62^. For the ten distinct cases of three survived species among five, the coexistence states with such a non-sub-cyclic structure are stable. Interestingly, the conventional coexistence states with a sub-cyclic structure among the three surviving species are unstable (See Supplementary Information for more details). It is also apparent that, dynamically, the coexistence states are not supported by spiral wave patterns.

### Role of intraspecific competition in promoting diverse coexistence states - a qualitative understanding

To understand the effect of intraspecific competition on coexistence qualitatively, we investigate the population change for each species *i* as a result of interspecific and intraspecific competitions as well as reproduction at time *t*, denoted as *C*_*i*_(*t*), *I*_*i*_(*t*) and *R*_*i*_(*t*) (scaled by the lattice size *N*), respectively. From the fact that the populations are determined by the balance between reproduction and competition, we find it useful to define two quantities: *H*_*i*_(*t*) = *R*_*i*_(*t*) − *C*_*i*_(*t*) and *S*_*i*_(*t*) = *R*_*i*_(*t*) − *C*_*i*_(*t*) − *I*_*i*_(*t*). We then have *H*_*i*_(*t*) − *S*_*i*_(*t*) = *I*_*i*_(*t*) ≥ 0 and the population at time *t* can be written as ${P}_{i}(t)={P}_{i}^{0}+{\int }_{0}^{t}\,{S}_{i}(k)dk$ for a given initial population ${P}_{i}^{0}$. For *S*_*i*_(*t*) > 0, species *i* can survive as its population tends to increase with time, which will cause a decrease in the population of the next species in the cycle (prey of species *i*) as a result of interspecific competitions, leading to possible extinction. For *S*_*i*_(*t*) < 0, the population of species *i* decreases and possibly becomes extinct. These simple observations imply that, in order for multiple species to survive, *S*_*i*_(*t*) must fluctuate about zero. Equivalently, for surviving species *i*, its population fluctuations can be described by a normal diffusion process: $\langle {S}_{i}^{2}(t)\rangle  \sim t$.

**Figure 6:**
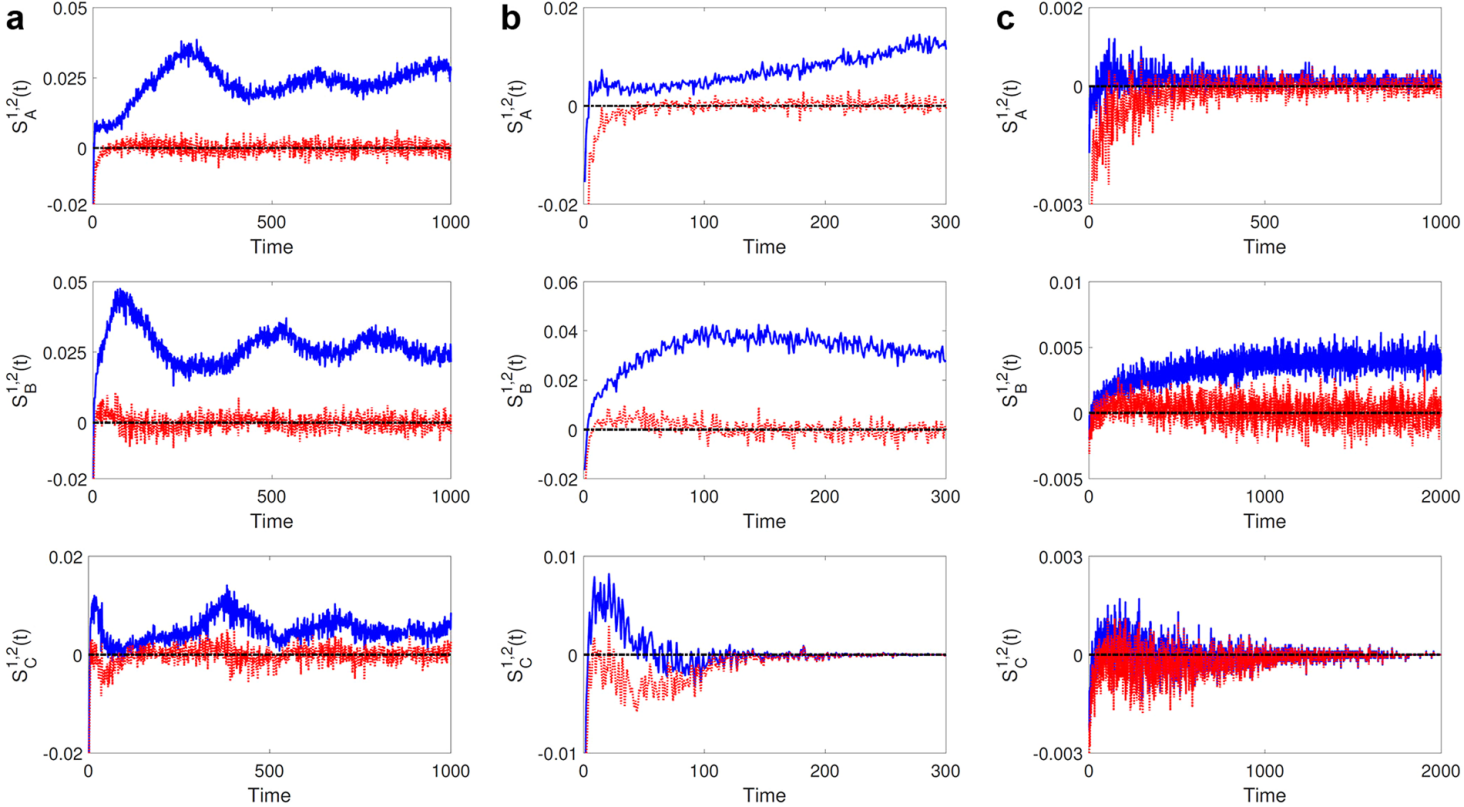
Qualitative indicator of effect of intraspecific competition on coexistence in RPS game. Each column shows the quantities *H*_*i*_ (blue) and *S*_*i*_ (red) for each species, where the corresponding coexistence states are indicated by three, two and one from left to right. (**a**) There is a gap between blue and red quantities all time. (**b**) The gap between the two quantities of a species (bottom panel) is reduced and finally disappears. The top and bottom panels of the third column in (**c**) show the collapsed gap between the two quantities.

For the case of the coexistence of three species in the RPS system, Fig. 6(a) shows the time evolution of *H*_*i*_(*t*) and *S*_*i*_(*t*). We observe that *H*_*i*_(*t*) > 0 but *S*_*i*_(*t*) fluctuates about zero, which indicates that, without intraspecific competitions, the corresponding populations tend to increase with time due to *H*_*i*_(*t*) > 0. However, intraspecific competition can reduce the populations, because *S*_*i*_ ≈ 0. This implies that a possible balance between the increasing and decreasing trends can be attained, stabilizing the populations. For the case of species extinction [Fig. 6(b,c)], we find that *H*_*i*_ can be positive initially but becomes negative due to the decrease in the reproduction as a result of strong intraspecific competitions.

For the cyclic system of three species (*A* → *B* → *C* → *A*), we note that each pair of species constitutes a predator-prey system. To examine the process leading to the coexistence of two species, we assume a decrease in the population of predator (*A*) from its initial population due to strong intraspecific competitions, which will immediately reduce the interspecific competition with its prey (*B*). As a result, the prey population can increase due to reproduction. The change in the population of species (*B*) will in turn enhance the interspecific competition with its prey (*C*), leading to a decrease in its population and possibly to its extinction. This chain of interactions indicates that intraspecific competition of a species can have a dramatic effect on the populations of other interacting species, potentially generating a distinct equilibrium state. To provide theoretical support, we identify the critical level of intraspecific competition leading to qualitative changes in the species populations through a mathematical analysis of the existence and the stability conditions of the equilibrium states listed in Table 1 (see Methods and Supplementary Information).

**Table 1:** Existence and stability conditions of fixed points of type **p**_2_ in the RPS game.

Species	*AB*	*AC*	*BC*
Fixed point	*w*_1_(*p*_*b*_, *p*_*a*_ − 2, 0)	*w*_2_(*p*_*c*_ − 2, 0, *p*_*a*_)	*w*_3_(0, *p*_*c*_, *p*_*b*_ − 2)
*λ*_*i*_	*w*_1_(*p*_*a*_(*p*_*b*_ − 2) + 4)/2	*w*_2_(*p*_*c*_(*p*_*a*_ − 2) + 4)/2	*w*_3_(*p*_*b*_(*p*_*c*_ − 2) + 4)/2
	−*w*_1_ *p*_*b*_(*p*_*a*_ − 2)/2	−*w*_2_ *p*_*a*_(*p*_*c*_ − 2)/2	−*w*_3_ *p*_*c*_(*p*_*b*_ − 2)/2
	−1	−1	−1
Existence	*p*_*a*_ > 2	*p*_*a*_ > 0	*p*_*b*_ > 2
	*p*_*b*_ > 0	*p*_*c*_ > 2	*p*_*c*_ > 0
	${p}_{b} > \frac{4-2{p}_{a}}{{p}_{a}+2}$	${p}_{a} > \frac{4-2{p}_{c}}{{p}_{c}+2}$	${p}_{c} > \frac{4-2{p}_{b}}{{p}_{b}+2}$
Stable Condition	*p*_*a*_(*p*_*b*_ − 2) + 4 < 0	*p*_*c*_(*p*_*a*_ − 2) + 4 < 0	*p*_*b*_(*p*_*c*_ − 2) + 4 < 0
	${p}_{a} > \frac{4}{2-{p}_{b}}$, *p*_*b*_ < 2	${p}_{c} > \frac{4}{2-{p}_{a}}$, *p*_*a*_ < 2	${p}_{b} > \frac{4}{2-{p}_{c}}$, *p*_*c*_ < 2

In the three species system, the coexistence state of two species is characteristic of that of a predator-prey system. Intuitively, the classic Lotka-Volterra model can be used to describe such a situation, where the populations can exhibit periodic oscillations with time (or a limit cycle in the phase space). We find that, however, in the presence of intraspecific competitions, the populations of the coexisting species do not exhibit periodic oscillations but steady states, as shown in Fig. 2(b). Thus, intraspecific competitions can either enhance coexistence and induce diverse coexistence states, or break the equilibrium and lead to extinction.

## Discussion

In the classic three- or five-species cyclic games, the species are on the equal footing in the sense that no particular species is superior or inferior to any other species. This intrinsic symmetry imposes a restriction on the survival or coexistence states of the system. For example, for the classic RPS game, either only one species survives as a result of interspecific interactions in which the end result is the disappearance of such competitions, or all three species can sustain and coexist^20–23^. For this reason the coexistence state of two species has never been reported before in the literature. Likewise, for the five-species RPSLS game, the known coexistence states contain a sole surviving species, three species, or all five species^52^. It is conceivable that, when the intrinsic symmetry among the competing species is broken, more diverse types of coexistence states can emerge.

Nonuniform intraspecific competitions represent one mechanism that can break the symmetry: they put the species on unequal footings. For example, if competitions among individuals in a species are stronger than those in another species, the former requires more resources to reproduce in order to survive and is therefore effectively “inferior” to the latter. As a result, a predator-prey type of behavior can emerge on the macroscopic scale where intraspecific competitions associated with the predator are stronger than those with the prey to reach a new equilibrium state in which the two surviving species are no longer on equal footing. Mathematically, it is not necessary for such a state to possess any intrinsic symmetry. The consequence is that coexistence states involving any number of species (insofar the number is less than or equal to the total number of species in the system) can arise.

The contributions of this paper are mathematical analyses, physical understanding, and comprehensive numerical tests that symmetry breaking can lead to more diverse coexistence states than previously reported. To accomplish this goal, we systematically studied three classes of cyclic game systems with either three or five species, subject to nonuniform, species dependent, intraspecific competitions. For each system, we focus on the asymptotic dynamical states (i.e., the coexistence states) of the system utilizing three approaches: ODE based stability analysis, microscopic Monte-Carlo simulation of the lattice model, and spatiotemporal evolution of the corresponding PDE model. A detailed bifurcation analysis of the ODE model reveals that, as the strength of the intraspecific competition for one species is systematically varied (while keeping the intraspecific competition strengths for the other species fixed), coexistence states of any number of species can arise in different parameter regimes. The occurrence of these states is further supported by both lattice and PDE simulations. A distinct feature is that the coexistence states here are not associated with any spiral wave patterns that were previously established as the underlying spatiotemporal dynamical structure supporting the coexistence of multiple species in cyclic game systems^22, 26^. Our findings suggest that symmetric breaking with nonuniform intraspecific competitions across the species may be more beneficial to biodiversity.

## Methods

### Numerical methods

All PDE models are solved by the standard spectral method and lattice simulations are of the Monte Carlo type. Lattice sizes vary from 100 × 100 to 500 × 500.

### Stability analysis of RPS game

In the classic RPS game in the absence of intraspecific competitions (i.e., *p*_*i*_ = 0), there are two possible states: one in which all three species coexist and another with only one surviving species. In the presence of intraspecific competitions, a new type of states in which two species coexist can arise, which can be seen by finding the fixed points of the ODE model, Eq. (2), and analyzing their stabilities. The first type of fixed points, denoted by **p**_1_, correspond to an extinction state:

4 $$(\frac{2}{2+{p}_{a}},\mathrm{0,}\,0),\,(0,\frac{2}{2+{p}_{b}},0),\,(0,\,0,\frac{2}{2+{p}_{c}}).$$

The second type **p**_2_ is for states in which two species coexist and one species is extinct:

5 $${w}_{1}\,({p}_{b},{p}_{a}-2,0),$$

6 $${w}_{2}\,({p}_{c}-2,0,{p}_{a}),$$

7 $${w}_{3}\,\mathrm{(0,}\,{p}_{c},{p}_{b}-2),$$

where *w*_1_ = 2/(*p*_*a*_ *p*_*b*_ + 2(*p*_*a*_ + *p*_*b*_) − 4), *w*_2_ = 2/(*p*_*a*_ *p*_*c*_ + 2(*p*_*a*_ + *p*_*c*_) − 4), and *w*_3_ = 2/(*p*_*b*_ *p*_*c*_ + 2(*p*_*b*_ + *p*_*c*_) − 4). The existence and stability conditions of type **p**_2_ fixed points are summarized in Table 1. The last type **p**_3_ corresponds to the state in which all three species survive, i.e., (a*, b*, c*), where

8 $${a}^{\ast }=\mathrm{2(}{p}_{b}({p}_{c}-\mathrm{2)}+\mathrm{4)}/{\rm{\Gamma }},\,{b}^{\ast }=\mathrm{2(}{p}_{c}({p}_{a}-\mathrm{2)}+\mathrm{4)}/{\rm{\Gamma }},\,{c}^{\ast }=\mathrm{2(}{p}_{a}({p}_{b}-\mathrm{2)}+\mathrm{4)}/{\rm{\Gamma }},$$

and Γ = *p*_*a*_ *p*_*b*_ *p*_*c*_ + 8 + 2[*p*_*a*_ *p*_*b*_ + *p*_*b*_ *p*_*c*_ + *p*_*c*_ *p*_*a*_ − 2(*p*_*a*_ + *p*_*b*_ + *p*_*c*_) + 12]. For a more detailed analysis of stability and existence of fixed points, see Supplementary Information.

To assess the stabilities of these different types of fixed points in a concrete way, we fix (*p*_*b*_, *p*_*c*_) = (1, 0.5) and vary the parameter *p*_*a*_. For *p*_*a*_ ≥ 0, there are three fixed points of type **p**_1_ and one fixed point of type **p**_3_. However, for *p*_*a*_ ≥ 2, only one fixed point of type **p**_2_ [Eq. (5)] exists. The three fixed points of type **p**_1_ are saddles for *p*_*a*_ ≥ 0. While for 0 < *p*_*a*_ ≤ 1.5 all fixed points are unstable, and there is an asymptotically stable heteroclinic cycle constituting three heteroclinic orbits connecting any two saddle fixed points (all of the **p**_1_ type). Since the cycle is arbitrarily close to the saddle fixed points, a small perturbation can cause a trajectory to diverge from the cycle, leading to extinction. For 1.5 < *p*_*a*_ ≤ 4, the fixed point of the type **p**_3_ [Eq. (8)] becomes stable, in which all species coexist. We note that, for *p*_*a*_ ≥ 2, fixed points of the type **p**_2_ [Eq. (5)] are created and are unstable. For *p*_*a*_ > 4, the fixed points given by Eqs (5) and (8) become stable and unstable, respectively, indicating the emergence of the coexistence state of two species. In this case, the fixed point given by Eq. (5) is globally stable. As *p*_*a*_ is increased further, this fixed point approaches an extinction state:

$$\frac{2}{{p}_{a}{p}_{b}+\mathrm{2(}{p}_{a}+{p}_{b})-4}\,({p}_{b},{p}_{a}-2,\mathrm{0)}\to \mathrm{(0},2/3,0)\,{\rm{as}}\,{p}_{a}\to \infty .$$

Figure 3(a) presents a bifurcation diagram of these behaviors.

## Electronic supplementary material

Supplementary information
