# Supplementary material for: Emergence of unusual coexistence states in cyclic game systems
Source: Sci Rep. 2017 Aug 7;7:7465. doi: 10.1038/s41598-017-07911-4 (PMC5547111; doi:10.1038/s41598-017-07911-4)
Supplement: Supplementary file 1 — Supplementary information [file 41598_2017_7911_MOESM1_ESM.pdf]

Supplementary Information for

Emergence of unusual coexistence states in cyclic game systems

Junpyo Park[1], Younghae Do[2], Bongsoo Jang[1], and Ying-Cheng Lai[3]

[1]Department of Mathematical Sciences,
Ulsan National Institute of Science and Technology,
Ulsan 44919, Republic of Korea
[2]Department of Mathematics, KNU-Center for Nonlinear Dynamics,
Kyungpook National University, Daegu 41566, Republic of Korea
[3]School of Electrical, Computer, and Energy Engineering,
Arizona State University, Tempe, Arizona 85287, USA

Contents

1 Stability analysis of rock-paper-scissors system **2**
1.1 Uniform intraspecific competitions 2
1.2 Nonuniform intraspecific competitions 2

2 Extended rock-paper-scissors system **5**
2.1 ODE based stability analysis 5
2.2 PDE model 7

3 Rock-paper-scissors-lizard-spock system **7**
3.1 ODE based Stability analysis 8
3.2 PDE model 10

4 Supplementary Tables **11**

5 Supplementary References **12**

6 Supplementary Figures **13**

1 Stability analysis of rock-paper-scissors system

1.1 Uniform intraspecific competitions

For uniform intraspecific competitions: $p_a = p_b = p_c = p$, the ODE model of the RPS system is given by

$$\begin{aligned} \frac{da}{dt} &= a\left[\mu(1-\rho) - \sigma c - \frac{p}{2}a\right], \\ \frac{db}{dt} &= b\left[\mu(1-\rho) - \sigma a - \frac{p}{2}b\right], \\ \frac{dc}{dt} &= c\left[\mu(1-\rho) - \sigma b - \frac{p}{2}c\right]. \end{aligned} \tag{S1}$$

There are three types of possible states: extinction (type $\mathbf{p}_1$), coexistence of two species (type $\mathbf{p}_2$), and coexistence of all three species (type $\mathbf{p}_3$). The corresponding fixed point solutions are listed in Supplementary Tab. S1. To be concrete, we set $\sigma = 1$ and $\mu = 1$. The system has three fixed points of type $\mathbf{p}_1$, which are unstable for $p > 0$. For the $\mathbf{p}_2$ type, three fixed points exist for $p > 2$ and $p^2 + 4p - 4 > 0$ but they are unstable with a positive eigenvalue $\lambda_2 > 0$, as listed in Supplementary Tab. S1. For $\mathbf{p}_3$, an unstable fixed point exists for $p < 1$ but it becomes stable for $p > 1$.

For $0 < p < 1$, the three fixed points of type $\mathbf{p}_1$ constitute a heteroclinic cycle, whose stability can be evaluated [S1]. Since they have identical eigenvalues, the corresponding eigenvalue ratios are identical as well

$$V_i = -\lambda_s^1/\lambda_u = \frac{2-p}{p}. \tag{S2}$$

The product V of the ratios is

$$V = \prod_{i=1}^{3} V_i = \left(\frac{2-p}{p}\right)^3 > 1. \tag{S3}$$

The heteroclinic cycle signifies coexistence but it is physically unstable as random perturbations will land the system into one of the extinction states. For $p \geq 1$, a typical trajectory converges to the stable fixed point $\mathbf{p}_3$. Supplementary Fig. S1 shows a bifurcation diagram and representative trajectories of the system.

1.2 Nonuniform intraspecific competitions

With nonuniform intraspecific competitions, the system becomes

$$\begin{aligned} \frac{da}{dt} &= a\left[(1-\rho) - c - \frac{p_a}{2}a\right], \\ \frac{db}{dt} &= b\left[(1-\rho) - a - \frac{p_b}{2}b\right], \\ \frac{dc}{dt} &= c\left[(1-\rho) - b - \frac{p_c}{2}c\right]. \end{aligned} \tag{S4}$$

There are three types of fixed points:

$$\mathbf{p}_1 : \left(\frac{2}{2+p_a}, 0, 0\right), \left(0, \frac{2}{2+p_b}, 0\right), \left(0, 0, \frac{2}{2+p_c}\right), \tag{S5}$$

$$\mathbf{p}_2 : w_4(p_b, p_a - 2, 0), w_5(p_c - 2, 0, p_a), w_6(0, p_c, p_b - 2), \tag{S6}$$

$$\mathbf{p}_3 : (a^*, b^*, c^*), \tag{S7}$$

where

$$
\begin{aligned}
w_4 &= 2/(p_a p_b + 2(p_a + p_b) - 4), \\
w_5 &= 2/(p_a p_c + 2(p_a + p_c) - 4), \\
w_6 &= 2/(p_b p_c + 2(p_b + p_c) - 4), \\
a^* &= 2(p_b(p_c - 2) + 4)/\Gamma, \\
b^* &= 2(p_c(p_a - 2) + 4)/\Gamma, \\
c^* &= 2(p_a(p_b - 2) + 4)/\Gamma, \\
\Gamma &= p_a p_b p_c + 2(p_a p_b + p_b p_c + p_c p_a) - 4(p_a + p_b + p_c) + 32.
\end{aligned}
$$

The three fixed points of type $\mathbf{p}_1$ are unstable:

$$
\lambda_1 = -1, \quad \lambda_2 = \frac{p_a - 2}{2 + p_a}, \quad \lambda_3 = \frac{p_a}{2 + p_a}.
$$

We summarize the existence and stability conditions of the three fixed points of type $\mathbf{p}_2$ in Supplementary Tab. S2.

To study the effect of nonuniform intraspecific competitions on coexistence, we consider three concrete scenarios: (1) fixing the values of the intraspecific competition rate for two species and varying the third, (2) fixing one rate and varying the remaining two, and (3) fixing the sum of the three rates ($p_1 + p_2 + p_3 = \alpha$) and varying the value of the sum. For the first scenario, for fixed $p_a = p_b$ and value of p_c open, we find coexistence states. In particular, for $p_a = p_b < 2$, as p_c is increased, global attractors such as stable heteroclinic cycles can arise, as well as coexisting state $\mathbf{p}_3$ of all three species and the two-species coexistence state $\mathbf{p}_2$, as shown in Supplementary Fig. S2. In this case, the existence range and the stabilities of the fixed points of types $\mathbf{p}_2$ and $\mathbf{p}_3$ depend on the value of p_c. If we decrease the value of $p_a = p_b$, the existence interval of the fixed point $\mathbf{p}_3$ as a globally stable attractor strictly increases, but that of the stable heteroclinic cycle decreases. For sufficiently large values of p_c, a fixed point of type $\mathbf{p}_2$ exists and is stable. That is, two species can coexist, which is not possible when the intraspecific competitions are uniform. For $p_a = p_b > 2$, coexistence of all three species is stable for any value of p_c.

For the second scenario, we fix the value of p_a and vary p_b and p_c. Supplementary Fig. S3 shows the emergence and disappearance of various survival states. Specifically, we observe a decrease in the parameter region for a stable heteroclinic cycle and one of fixed points of type $\mathbf{p}_2$ (AC), but the region for the survival state of species B and C does not change, as detailed in Supplementary Tab. S2. That is, fixed points of type $\mathbf{p}_2$ have emerged. We also observe that for an increased value of p_a, varying p_b and p_c can expand the parameter regions for the fixed point $\mathbf{p}_3$. In general, nonuniform intraspecific competitions can induce new survival states and/or disappearance of previous survival states.

For the third scenario, we vary p_a, p_b, $p_c \geq 0$ while keeping their sum α fixed. Supplementary Fig. S4 shows the various survival states in the 2-simplex plane for several α values ranging from 0.6 to 60. We use different colors to denote different states: a stable heteroclinic cycle (white), three different fixed points of type $\mathbf{p}_2$ (red, blue and yellow for coexisting species AB, AC and BC, respectively), and the fixed point $\mathbf{p}_3$ for stable coexistence of all species (black). Specifically, for $\alpha < 2$, a stable heteroclinic cycle constituting three saddle fixed points of type $\mathbf{p}_1$ is the global attractor of the system, whereas fixed points of type $\mathbf{p}_2$ do not exist and the fixed point $\mathbf{p}_3$ exists but it is unstable. For $\alpha > 2$, a dramatic change in the survival states occurs: depending on the rates of the intraspecific competitions, a coexisting state of two species emerges, which has not been observed in previous studies of the RPS system. In general, the coexistence states $\mathbf{p}_2$

and $\mathbf{p}_3$ begin to emerge from the three vertices of the 2-simplex plane $p_a + p_b + p_c = \alpha$. As α is increased, the parameter region of $\mathbf{p}_3$ expands toward the center of the 2-simplex from each of the three vertices and merge into a larger region, as can be seen in the top two rows in Supplementary Fig. S4. The two-species coexistence states $\mathbf{p}_2$ are born from the vertices of the 2-simplex and expand following its edges. From the bottom two rows in Supplementary Fig. S4, we see that, for a sufficiently large value of α, the interior of the 2-simplex is fully covered by the two distinct coexistence states $\mathbf{p}_2$ and $\mathbf{p}_3$. For $\alpha \geq \alpha_h \approx 3.7$, the stable heteroclinic cycle no longer exists. As the value of α is increased further, the ratio of the area of three basins for $\mathbf{p}_2$ to the total area of the 2-simplex plane increases until when $\alpha \leq \alpha_c \approx 5.9$ and then decreases for $\alpha > \alpha_c$.

We numerically calculate the existence boundaries for each attractor for $\alpha = 5$ with parameters p_a, p_b and p_c from the 2-simplex plane, as shown in Supplementary Fig. S5. Exploiting the existence condition of the fixed points of type $\mathbf{p}_2$ as listed in Supplementary Tab. S2, we can derive each boundary curve analytically. For example, setting $p_b = 4/(2 - p_c)$, we can obtain the parameter boundary of the coexisting state of two species (B and C - yellow region in Supplementary Fig. S5). In particular, we have

$$p_a + \frac{4}{2 - p_c} + p_c = \alpha \quad (\alpha > 2),$$

which implies

$$p_a = \alpha - \frac{4}{2 - p_c} - p_c = \alpha - \frac{p_c^2 - 2p_c - 4}{p_c - 2}.$$

To further study the difference in the characteristics between coexistence of two and three species, we study the dynamical behaviors of the system for two parameter settings located above and below the boundary in Supplementary Fig. S5: (1) $(p_a, p_b, p_c) = (1.7, 2.8, 0.5)$ for $\mathbf{p}_2$ and (2) $(p_a, p_b, p_c) = (1.7, 2.7, 0.6)$ for $\mathbf{p}_3$. We see that species A becomes extinct under parameter setting (1), while it survives under setting (2). To understand this behavior, we note that, for setting (1), although the intraspecific competitions within A and B are stronger than that of species C, it is A that becomes extinct. Supplementary Fig. S6 shows the densities of the three species from the lattice simulation, and we obtain essentially the same patterns from the corresponding PDE model (data not shown). How interspecific and intraspecific competitions as well as reproduction affect the densities can also be seen from Supplementary Fig. S6.

A possible dynamical process leading to the coexistence of two species is as follows. A decrease in the population of species B subject to strong intraspecific competitions can be beneficial to its prey (species C), leading to an increase in the prey population. As a result, species A will be at a disadvantage because it is the prey of C. It can happen that reproduction of A is not sufficient to sustain the population decrease due to interactions with C, leading to extinction of A. That is, an increase in self-competition within one species can cause the extinction of other species.

For parameter setting (2), species A survives even if its population is much reduced, as shown in Supplementary Fig. S7. This behavior is quite different from that under setting (1), indicating the emergence of a critical borderline between the two settings. This may have implications for population control. For example, under setting (2), intraspecific competitions within species B are stronger than those within the other two species, leading to an increase in their populations, which in turn causes their predators' populations to increase.

2 Extended rock-paper-scissors system

For the five-species, extended rock-paper-scissors (ERPS) system, in the absence of intraspecific competitions, there are three possible states [S2]: the coexistence state of all five species, states in which two non-interacting species survive, and extinction states of only one surviving species. As demonstrated in the main text, with nonuniform intraspecific competitions all possible coexistence states can arise.

2.1 ODE based stability analysis

The ODE model of the ERPS system is

$$\begin{aligned}
\frac{da}{dt} &= a\left[\mu(1-\rho) - \sigma e - \frac{p_a}{2}a\right], \\
\frac{db}{dt} &= b\left[\mu(1-\rho) - \sigma a - \frac{p_b}{2}b\right], \\
\frac{dc}{dt} &= c\left[\mu(1-\rho) - \sigma b - \frac{p_c}{2}c\right], \\
\frac{dd}{dt} &= d\left[\mu(1-\rho) - \sigma c - \frac{p_d}{2}d\right], \\
\frac{de}{dt} &= e\left[\mu(1-\rho) - \sigma d - \frac{p_e}{2}e\right].
\end{aligned} \tag{S8}$$

To be concrete, we fix $\sigma = \mu = 1$. There are five types of fixed points. The first type, denoted by $\mathbf{q}_1$, corresponds to various extinction states:

$$\begin{aligned}
&\left(\frac{2}{2+p_a}, 0, 0, 0, 0\right), \left(0, \frac{2}{2+p_b}, 0, 0, 0\right), \left(0, 0, \frac{2}{2+p_c}, 0, 0\right), \\
&\left(0, 0, 0, \frac{2}{2+p_d}, 0\right), \left(0, 0, 0, 0, \frac{2}{2+p_e}\right).
\end{aligned} \tag{S9}$$

The second type $\mathbf{q}_2$ specifies states in which only two species coexist. Five of such fixed points are

$$\frac{2}{p_a p_c + 2(p_a + p_c)}(p_c, 0, p_a, 0, 0), \tag{S10}$$

$$\frac{2}{p_a p_d + 2(p_a + p_d)}(p_d, 0, 0, p_a, 0), \tag{S11}$$

$$\frac{2}{p_b p_d + 2(p_b + p_d)}(0, p_d, 0, p_b, 0), \tag{S12}$$

$$\frac{2}{p_b p_e + 2(p_b + p_e)}(0, p_e, 0, 0, p_b), \tag{S13}$$

$$\frac{2}{p_c p_e + 2(p_c + p_e)}(0, 0, p_e, 0, p_c), \tag{S14}$$

which are unstable. The third type $\mathbf{q}_3$ signifies the coexistence state of three species. The possibly stable fixed points of this type are

$$\frac{2(p_bp_d, p_d(p_a-2), 0, p_ap_b, 0)}{p_ap_bp_d + 2(p_ap_b + p_ap_d + p_bp_d) - 4p_d}, \tag{S15}$$

$$\frac{2(p_cp_d, 0, p_ap_d, p_a(p_c-2), 0)}{p_ap_cp_d + 2(p_ap_c + p_ap_d + p_cp_d) - 4p_a}, \tag{S16}$$

$$\frac{2(p_c(p_e-2), 0, p_ap_e, 0, p_ap_c)}{p_ap_cp_e + 2(p_ap_c + p_ap_e + p_cp_e) - 4p_c}, \tag{S17}$$

$$\frac{2(0, p_cp_e, p_e(p_b-2), 0, p_bp_c)}{p_bp_cp_e + 2(p_bp_c + p_bp_e + p_cp_e) - 4p_e}, \tag{S18}$$

$$\frac{2(0, p_dp_e, 0, p_bp_e, p_b(p_d-2))}{p_bp_dp_e + 2(p_bp_d + p_bp_e + p_dp_e) - 4p_b}. \tag{S19}$$

The fourth type $\mathbf{q}_4$ describes the state in which four species coexist and only one species is extinct. For example, one such fixed point, denoted as $(a^*, 0, c^*, d^*, e^*)$, is given by

$$a^* = \frac{2(p_c(p_d(p_e-2)+4)-8)}{\Sigma_4}, \quad c^* = \frac{p_ap_dp_e}{\Sigma_4}, \quad d^* = \frac{2p_ap_e(p_c-2)}{\Sigma_4}, \quad e^* = \frac{2p_a(p_c(p_d-2)+4)}{\Sigma_4}, \tag{S20}$$

where

$$\Sigma_4 = p_ap_cp_dp_e + 2(p_ap_cp_d + p_ap_cp_e + p_ap_dp_e + p_cp_dp_e) - 4(p_ap_c + p_ap_e + p_cp_d) + 8(p_a + p_c) - 16.$$

The last type $\mathbf{q}_5$ corresponds to the state in which all five species survive, i.e., $(a^*, b^*, c^*, d^*, e^*)$, where

$$\begin{aligned} a^* &= \frac{2(p_bp_cp_dp_e - 2p_bp_cp_d + 4p_bp_c - 8p_b + 16)}{\Sigma_5}, \\ b^* &= \frac{2(p_ap_cp_dp_e - 2p_cp_dp_e + 4p_cp_d - 8p_c + 16)}{\Sigma_5}, \\ c^* &= \frac{2(p_ap_bp_dp_e - 2p_ap_dp_e + 4p_dp_e - 8p_d + 16)}{\Sigma_5}, \\ d^* &= \frac{2(p_ap_bp_cp_e - 2p_ap_bp_e + 4p_ap_e - 8p_e + 16)}{\Sigma_5}, \\ e^* &= \frac{2(p_ap_bp_cp_d - 2p_ap_bp_c + 4p_ap_b - 8p_a + 16)}{\Sigma_5}, \end{aligned} \tag{S21}$$

and

$$\begin{aligned} \Sigma_5 &= p_ap_bp_cp_dp_e + 2(p_ap_bp_cp_d + p_ap_bp_cp_e + p_ap_bp_dp_e + p_ap_cp_dp_e + p_bp_cp_dp_e) \\ &- 4(p_ap_bp_c + p_ap_bp_e + p_bp_cp_d + p_ap_dp_e + p_cp_dp_e) + 8(p_ap_b + p_bp_c + p_ap_e + p_cp_d + p_dp_e) \\ &- 16(p_a + p_b + p_c + p_d + p_e) + 192. \end{aligned}$$

To assess the stabilities of the fixed points, we fix $(p_a, p_b, p_d, p_e) = (1.9, 2, 1.3, 0.7)$ and vary the parameter p_c. For $p_c > 0$, there are fixed points of types $\mathbf{q}_1$ and $\mathbf{q}_2$ which are unstable. For $p_c > 2$, the fixed point (S16) of type $\mathbf{q}_3$ can exist and the fixed point (S20) of type $\mathbf{q}_4$ emerges for $3.5 \leq p_c < 5.8$. For $0 < p_c < 2.6$,

although the fixed points of types $\mathbf{q}_1$, $\mathbf{q}_2$, $\mathbf{q}_3$ and $\mathbf{q}_5$ exist, only the cycles consisting of the heteroclinic orbits connecting saddle fixed points of type $\mathbf{q}_2$ are numerically observable. For $2.6 \leq p_c < 5.6$, the fixed point $\mathbf{q}_5$ becomes stable in which all species coexist. However, this fixed point no longer exists for $p_c \geq 5.6$. For $p_c \geq 5.8$, the fixed points (S20) disappear. For $p_c \geq 5.8$, the three-species coexisting fixed points (S16) become stable and approach the two-species fixed points (S11) as $p_c \to \infty$. Thus, for large values of p_c, the coexistence states of two species are numerically observable, as shown in the bifurcation diagram and a spatial snapshot (Figs. 3 and 4, respectively) in the main text. In general, as the intraspecific competition parameter is increased, a variety of coexistence states can emerge.

2.2 PDE model

Taking into account the spatial dimension, we obtain a set of PDEs:

$$\begin{aligned}
\frac{\partial a(\mathbf{x},t)}{\partial t} &= M\Delta a(\mathbf{x},t) + \mu a(\mathbf{x},t)[1-\rho(\mathbf{x},t)] - \sigma e(\mathbf{x},t)a(\mathbf{x},t) - \frac{p_a}{2}a(\mathbf{x},t)a(\mathbf{x},t), \\
\frac{\partial b(\mathbf{x},t)}{\partial t} &= M\Delta b(\mathbf{x},t) + \mu b(\mathbf{x},t)[1-\rho(\mathbf{x},t)] - \sigma a(\mathbf{x},t)b(\mathbf{x},t) - \frac{p_b}{2}b(\mathbf{x},t)b(\mathbf{x},t), \\
\frac{\partial c(\mathbf{x},t)}{\partial t} &= M\Delta c(\mathbf{x},t) + \mu c(\mathbf{x},t)[1-\rho(\mathbf{x},t)] - \sigma b(\mathbf{x},t)c(\mathbf{x},t) - \frac{p_c}{2}c(\mathbf{x},t)c(\mathbf{x},t), \qquad \text{(S22)} \\
\frac{\partial d(\mathbf{x},t)}{\partial t} &= M\Delta d(\mathbf{x},t) + \mu d(\mathbf{x},t)[1-\rho(\mathbf{x},t)] - \sigma c(\mathbf{x},t)d(\mathbf{x},t) - \frac{p_d}{2}d(\mathbf{x},t)d(\mathbf{x},t), \\
\frac{\partial e(\mathbf{x},t)}{\partial t} &= M\Delta e(\mathbf{x},t) + \mu e(\mathbf{x},t)[1-\rho(\mathbf{x},t)] - \sigma d(\mathbf{x},t)e(\mathbf{x},t) - \frac{p_e}{2}e(\mathbf{x},t)e(\mathbf{x},t),
\end{aligned}$$

where $a(\mathbf{x},t)$, $b(\mathbf{x},t)$, $c(\mathbf{x},t)$, $d(\mathbf{x},t)$, and $e(\mathbf{x},t)$ denote the densities of the five species at site $\mathbf{x} = (x_1, x_2)$ and time t on a square domain with periodic boundary conditions.

3 Rock-paper-scissors-lizard-spock system

For the rock-paper-scissors-lizard-spock (RPSLS) system of five species, in the absence of intraspecific competitions, three types of distinct dynamical states can arise [S3] [S4]: the coexistence state of all five species, states in which three species having a sub-cyclic interacting structure coexist, and extinction states of only one surviving species. In the main text, we demonstrate that intraspecific competitions can induce coexistence states of any possible number of surviving species.

3.1 ODE based Stability analysis

Similar to the ERPS system, the coupled ODE model for the RPSLS system is

$$\begin{aligned}
\frac{da}{dt} &= a\left[\mu(1-\rho) - \sigma e - \sigma c - \frac{p_a}{2}a\right], \\
\frac{db}{dt} &= b\left[\mu(1-\rho) - \sigma a - \sigma d - \frac{p_b}{2}b\right], \\
\frac{dc}{dt} &= c\left[\mu(1-\rho) - \sigma b - \sigma e - \frac{p_c}{2}c\right], \\
\frac{dd}{dt} &= d\left[\mu(1-\rho) - \sigma c - \sigma a - \frac{p_d}{2}d\right], \\
\frac{de}{dt} &= e\left[\mu(1-\rho) - \sigma d - \sigma b - \frac{p_e}{2}e\right].
\end{aligned} \tag{S23}$$

There are different types of fixed points with different numbers of surviving species. For $\sigma = \mu = 1$, fixed points of different types are listed below.

The first type, denoted as $\mathbf{r}_1$, describes the extinction states:

$$\begin{aligned}
&\left(\frac{2}{2+p_a}, 0, 0, 0, 0\right), \left(0, \frac{2}{2+p_b}, 0, 0, 0\right), \left(0, 0, \frac{2}{2+p_c}, 0, 0\right), \\
&\left(0, 0, 0, \frac{2}{2+p_d}, 0\right), \left(0, 0, 0, 0, \frac{2}{2+p_e}\right).
\end{aligned} \tag{S24}$$

The second type $\mathbf{r}_2$ represents states in which only two species survive. For example, two of the ten possible fixed points are

$$\frac{2(p_c-2, 0, p_a, 0, 0)}{p_a p_c + 2(p_a+p_c) - 4}, \text{ and } \frac{2(0, 0, p_d, p_c-2, 0)}{p_c p_d + 2(p_c+p_d) - 4}, \tag{S25}$$

which are unstable. The third type, denoted by $\mathbf{r}_3$, corresponds to three coexisting species. In this case, there are ten such fixed points, three of which are

$$\frac{2(0, p_c(p_d-2)+4, p_d(p_b-2)+4, p_b(p_c-2)+4, 0)}{\Delta_{31}}, \tag{S26}$$

$$\frac{2(0, 0, p_d(p_e-2)+4, p_e(p_c-2)+4, p_c(p_d-2)+4)}{\Delta_{32}}, \tag{S27}$$

and

$$\frac{2((p_c-2)(p_e-2), 0, p_a(p_e-2), 0, p_a p_c)}{\Delta_{33}}, \tag{S28}$$

where

$$\begin{aligned}
\Delta_{31} &= p_b p_c p_d + 2(p_b p_c + p_b p_d + p_c p_d) - 4(p_b + p_c + p_d) + 32, \\
\Delta_{32} &= p_c p_d p_e + 2(p_c p_d + p_c p_e + p_d p_e) - 4(p_d + p_e + p_c) + 32, \\
\Delta_{33} &= p_a p_c p_e + 2(p_a p_c + p_a p_e + p_c p_e) - 4(p_a + p_c + p_e) + 8.
\end{aligned}$$

Fixed points such as those in (S28) have a *non-sub-cyclic structure* and can be stable in certain parameter ranges, while other five points including (S26)-(S27) with a sub-cyclic structure are always unstable as the eigenvalues of the underlying Jacobian matrices under the existence condition are positive. For example, for

the fixed point (S26), we have

$$\begin{aligned}
\lambda_1 &= -1, \quad \lambda_2 = \frac{(p_b-2)(p_c(p_d-2)+4)}{\Delta_{31}}, \quad \lambda_3 = \frac{p_d(p_b(p_c-2)+4)}{\Delta_{31}}, \\
\lambda_{4,5} &= [128 - 80(p_c+p_d) + 8(p_c^2+p_d^2) + 56p_cp_d - 8p_cp_d(p_c+p_d) + 2p_c^2p_d^2 \\
&\quad -p_b(p_c(-56+52p_d-6p_d^2) + 8(10-7p_d+p_d^2) + p_c^2(8-6p_d+p_d^2)) \\
&\quad -p_b^2(-2(p_d-2)^2 + p_c(8-6p_d+p_d^2) + p_c^2(-2+p_d+p_d^2)) \\
&\quad \pm\{(\Delta_{31})^2(p_b^2(2-p_c+p_d)^2 + (p_d-2)(4p_c(p_d-2) + p_c^2(p_d-2) + 4(p_d+6)) \\
&\quad -2p_b(p_c^2(p_d-2) + p_c(8-6p_d+p_d^2) + 2(-4+4p_d+p_d^2)))\}^{\frac{1}{2}}]/(\Delta_{31})^2,
\end{aligned} \tag{S29}$$

and λ_3 is positive because of the inequality $p_b(p_c-2)+4>0$. An illustration of the classification of the structures of the fixed points of type $\mathbf{r}_3$ is presented in Supplementary Fig. S8.

The fourth fixed-point type $\mathbf{r}_4$ corresponds to states in which four species coexist and one species is extinct. One such fixed point, for example $(a^*, b^*, c^*, 0, e^*)$, is given by

$$\begin{aligned}
a^* &= \frac{2(p_bp_cp_e + 4(p_b+p_c+p_e) - 2p_b(p_c+p_e) - 8)}{\Delta_4} \\
b^* &= \frac{2(p_ap_cp_e + 4(p_c+p_e) - 2p_cp_e - 8)}{\Delta_4} \\
c^* &= \frac{2(p_e-2)(p_a(p_b-2)+4)}{\Delta_4}, \quad e^* = \frac{2p_c(p_a(p_b-2)+4)}{\Delta_4},
\end{aligned} \tag{S30}$$

where

$$\begin{aligned}
\Delta_4 &= p_ap_bp_cp_e + 2(p_ap_bp_c + p_ap_bp_e + p_ap_cp_e + p_bp_cp_e) \\
&- 4(p_ap_b + p_ap_c + p_bp_c + p_ap_e + p_bp_e + p_cp_e) + 32(p_c+p_e) + 8(p_a+p_b) - 64.
\end{aligned}$$

The last fixed-point type, $\mathbf{r}_5$, characterizes the coexistence state of all five species, which is given by $(a^*, b^*, c^*, d^*, e^*)/\Delta_5$, where

$$\begin{aligned}
a^* &= 2\{16 - 8(p_b+p_c+p_d) + 4(p_bp_c + p_bp_d + p_cp_d + p_dp_e) - 2(p_bp_cp_d + p_bp_dp_e) + p_bp_cp_dp_e\}, \\
b^* &= 2\{16 - 8(p_c+p_d+p_e) + 4(p_cp_d + p_ap_e + p_cp_e + p_dp_e) - 2(p_ap_cp_e + p_cp_dp_e) + p_ap_cp_dp_e\}, \\
c^* &= 2\{16 - 8(p_a+p_d+p_e) + 4(p_ap_b + p_ap_d + p_ap_e + p_dp_e) - 2(p_ap_bp_d + p_ap_dp_e) + p_ap_bp_dp_e\}, \\
d^* &= 2\{16 - 8(p_a+p_b+p_e) + 4(p_ap_b + p_bp_c + p_ap_e + p_bp_e) - 2(p_ap_bp_e + p_bp_cp_e) + p_ap_bp_cp_e\}, \\
e^* &= 2\{16 - 8(p_a+p_b+p_c) + 4(p_ap_b + p_ap_c + p_bp_c + p_cp_d) - 2(p_ap_bp_c + p_ap_cp_d) + p_ap_bp_cp_d\},
\end{aligned} \tag{S31}$$

and

$$\begin{aligned}
\Delta_5 &= p_ap_bp_cp_dp_e + 2(p_ap_bp_cp_d + p_ap_bp_cp_e + p_ap_bp_dp_e + p_ap_cp_dp_e + p_bp_cp_dp_e) \\
&- 4(p_ap_bp_c + p_ap_bp_d + p_ap_cp_d + p_bp_cp_d + p_ap_bp_e + p_ap_cp_e + p_bp_cp_e + p_ap_dp_e + p_bp_dp_e + p_cp_dp_e) \\
&+ 32(p_ap_b + p_bp_c + p_cp_d + p_ap_e + p_dp_e) + 8(p_ap_c + p_ap_d + p_bp_d + p_bp_e + p_cp_e) \\
&- 64(p_a+p_b+p_c+p_d+p_e) + 224.
\end{aligned}$$

To analyze the stabilities of all the fixed points, we set $(p_a, p_b, p_c, p_d) = (0.3, 1.1, 2.5, 0.7)$ and vary the

parameter p_e. For $p_e \geq 0$, there are five fixed points of type $\mathbf{r}_1$, fixed points (S25) of type $\mathbf{r}_2$, points (S26) and (S27) of type $\mathbf{r}_3$, and points (S31) of type $\mathbf{r}_5$. For $p_e \geq 2.1$, fixed points (S28) and (S30) of types $\mathbf{r}_3$ and $\mathbf{r}_4$, respectively, are created. For $0 < p_e < 1.8$, all existing fixed points of types $\mathbf{r}_1$, $\mathbf{r}_2$, $\mathbf{r}_3$, and $\mathbf{r}_5$ are unstable. We find a stable cycle consisting of heteroclinic orbits among saddle fixed points of types $\mathbf{r}_1$ and $\mathbf{r}_2$. For $1.8 < p_e < 2.4$, the type $\mathbf{r}_5$ exists as a stable attractor, while the other fixed points are always unstable. For $2.4 \leq p_e < 8$, the global coexistence state of five species no longer exists but the fixed point (S30) becomes stable in which four species coexist. For $p_e \geq 8$, the fixed points (S30) disappear and the fixed points (S28) become stable in which three species survive. Even though the fixed points (S28) are stable, they will converge to the fixed points (S25) of type $\mathbf{r}_2$ for $p_e \to \infty$. It is thus possible to observe a coexistence state of two species for large values of p_e. The results of the stability analysis and numerical simulations are illustrated in the bifurcation diagram and spatial snapshots in the main text (Figs. 3 and 5, respectively).

The extinction state (S24) can be obtained from a different parameter setting such as $(p_a, p_b, p_c, p_d) = (0.01, 1.1, 2.5, 0.7)$. By varying p_e, the stable fixed point (S28) converges to the extinction state of type $\mathbf{r}_1$ because of the simultaneous decrease in the populations of the two species C and E, as shown in Fig. 5 in the main text.

3.2 PDE model

Similarly to the ERPS system, we obtain the PDE model for the RPSLS game as

$$\begin{aligned}
\frac{\partial a(\mathbf{x},t)}{\partial t} &= M\Delta a(\mathbf{x},t) + \mu a(\mathbf{x},t)[1-\rho(\mathbf{x},t)] - \sigma e(\mathbf{x},t)a(\mathbf{x},t) \\
&\quad -\gamma a(\mathbf{x},t)c(\mathbf{x},t) - \frac{p_a}{2}a(\mathbf{x},t)a(\mathbf{x},t), \\
\frac{\partial b(\mathbf{x},t)}{\partial t} &= M\Delta b(\mathbf{x},t) + \mu b(\mathbf{x},t)[1-\rho(\mathbf{x},t)] - \sigma a(\mathbf{x},t)b(\mathbf{x},t) \\
&\quad -\gamma b(\mathbf{x},t)d(\mathbf{x},t) - \frac{p_b}{2}b(\mathbf{x},t)b(\mathbf{x},t), \\
\frac{\partial c(\mathbf{x},t)}{\partial t} &= M\Delta c(\mathbf{x},t) + \mu c(\mathbf{x},t)[1-\rho(\mathbf{x},t)] - \sigma b(\mathbf{x},t)c(\mathbf{x},t) \\
&\quad -\gamma c(\mathbf{x},t)e(\mathbf{x},t) - \frac{p_c}{2}c(\mathbf{x},t)c(\mathbf{x},t), \qquad \text{(S32)} \\
\frac{\partial d(\mathbf{x},t)}{\partial t} &= M\Delta d(\mathbf{x},t) + \mu d(\mathbf{x},t)[1-\rho(\mathbf{x},t)] - \sigma c(\mathbf{x},t)d(\mathbf{x},t) \\
&\quad -\gamma a(\mathbf{x},t)d(\mathbf{x},t) - \frac{p_d}{2}d(\mathbf{x},t)d(\mathbf{x},t), \\
\frac{\partial e(\mathbf{x},t)}{\partial t} &= M\Delta e(\mathbf{x},t) + \mu e(\mathbf{x},t)[1-\rho(\mathbf{x},t)] - \sigma d(\mathbf{x},t)e(\mathbf{x},t) \\
&\quad -\gamma b(\mathbf{x},t)e(\mathbf{x},t) - \frac{p_e}{2}e(\mathbf{x},t)e(\mathbf{x},t).
\end{aligned}$$

4 Supplementary Tables

Type	$\mathbf{p}_1$	$\mathbf{p}_2$	$\mathbf{p}_3$
Fixed point	$(w_1, 0, 0)$<br>$(0, w_1, 0)$<br>$(0, 0, w_1)$	$w_2(p, p - 2\sigma, 0)$<br>$w_2(p - 2\sigma, 0, p)$<br>$w_2(0, p, p - 2\sigma)$	$w_3(1, 1, 1)$
λ_i	$\lambda_1 = -1$<br>$\lambda_2 = \frac{p-2}{2+p}$<br>$\lambda_3 = \frac{p}{2+p}$	$\lambda_1 = -1$<br>$\lambda_2 = \frac{(p-1)^2+3}{p^2+4p-4}$<br>$\lambda_3 = \frac{p(2-p)}{p^2+4p-4}$	$\lambda_1 = -1$<br>$\lambda_2 = \frac{(1-p)\pm\sqrt{3}i}{8+p}$<br>$\lambda_3 = \lambda_2$
Existence	always	$p > 2\sigma$<br>$p^2 + 4\mu p - 4\mu\sigma > 0$	always
Stability	unstable	unstable	unstable, if $p < 1$<br>stable, if $p > 1$

Supplementary Table S1: Existence and stabilities of all existing fixed points of RPS system (S1). For uniform intraspecific competitions, the RPS system admits three types of fixed points: $w_1 = 2\mu/(2\mu + p)$, $w_2 = 2\mu/(p^2 + 4\mu p - 4\mu\sigma)$, and $w_3 = 2\mu/(6\mu + 2\sigma + p)$.

Species	AB	AC	BC
Fixed point	$w_4(p_b, p_a - 2, 0)$	$w_5(p_c - 2, 0, p_a)$	$w_6(0, p_c, p_b - 2)$
λ_i	$w_4(p_a(p_b - 2) + 4)/2$<br>$-w_4 p_b(p_a - 2)/2$<br>-1	$w_5(p_c(p_a - 2) + 4)/2$<br>$-w_5 p_a(p_c - 2)/2$<br>-1	$w_6(p_b(p_c - 2) + 4)/2$<br>$-w_6 p_c(p_b - 2)/2$<br>-1
Existence	$p_a > 2$<br>$p_b > 0$<br>$p_b > \frac{4-2p_a}{p_a+2}$	$p_a > 0$<br>$p_c > 2$<br>$p_a > \frac{4-2p_c}{p_c+2}$	$p_b > 2$<br>$p_c > 0$<br>$p_c > \frac{4-2p_b}{p_b+2}$
Stable Condition	$p_a(p_b - 2) + 4 < 0$<br>$p_a > \frac{4}{2-p_b}, p_b < 2$	$p_c(p_a - 2) + 4 < 0$<br>$p_c > \frac{4}{2-p_a}, p_a < 2$	$p_b(p_c - 2) + 4 < 0$<br>$p_b > \frac{4}{2-p_c}, p_c < 2$

Supplementary Table S2: Summary of existence and stability conditions of fixed points of type $\mathbf{p}_2$ for RPS system (S4). For nonuniform intraspecific competitions, the RPS system allows three different fixed points of type $\mathbf{p}_2$: $w_4 = 2/(p_a p_b + 2(p_a + p_b) - 4)$, $w_5 = 2/(p_a p_c + 2(p_a + p_c) - 4)$, and $w_6 = 2/(p_b p_c + 2(p_b + p_c) - 4)$ for $\sigma = 1$ and $\mu = 1$.

5 Supplementary References

[S1] Shaw, K. M., Park, Y.-M., Chiel, H. J. & Thomas, P. J. Phase resetting in an asymptotically phaseless system: on the phase response of limit cycles verging on a heteroclinic orbit. *Siam J. Appl. Dyn. Sys.* **11**, 350-391 (2012).

[S2] Feng, S.-S. & Qiang, C.-C. Self-organization of five species in a cyclic competition game. *Physica A* **392**, 4675-4682 (2013).

[S3] Vukov, J., Szolnoki, A. & Szabó, G. Diverging fluctuations in a spatial five-species cyclic dominance game. *Phys. Rev. E* **88**, 022123 (2013).

[S4] Zheng, H.-Y., Yao, N., Huang, Z.-G., Park, J., Do, Y. & Lai, Y.-C. Mesoscopic interactions and species coexistence in evolutionary game dynamics of cyclic competitions. *Sci. Rep* **4**, 7486 (2014).

6 Supplementary Figures

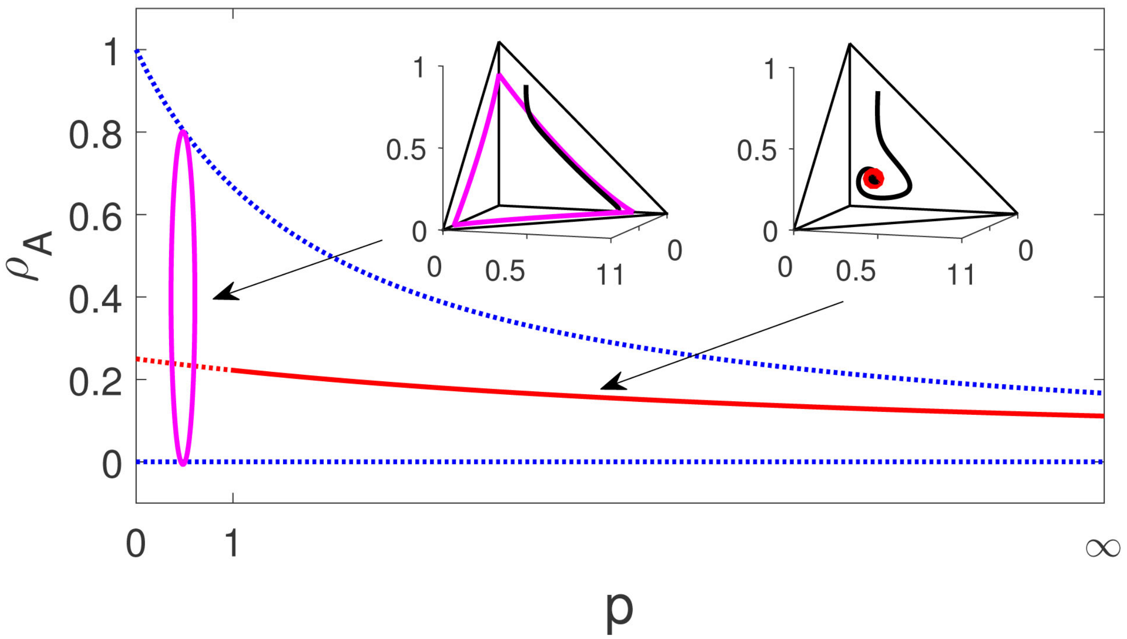

Supplementary Figure S1: Bifurcation diagram of the RPS system (S1). For $0 \leq p < 1$, an asymptotically stable heteroclinic cycle exists and the fixed point of type $\mathbf{p}_3$ in which all three species coexist is unstable (red dotted line). For $p > 1$, the heteroclinic cycle loses its stability and the fixed point $\mathbf{p}_3$ becomes stable (red straight line). Blue dotted lines represent the unstable fixed point $\mathbf{p}_1$. The insets illustrate the behaviors of a typical trajectory (black), the heteroclinic cycle (connected magenta lines), and the fixed point $\mathbf{p}_3$ (red dot).

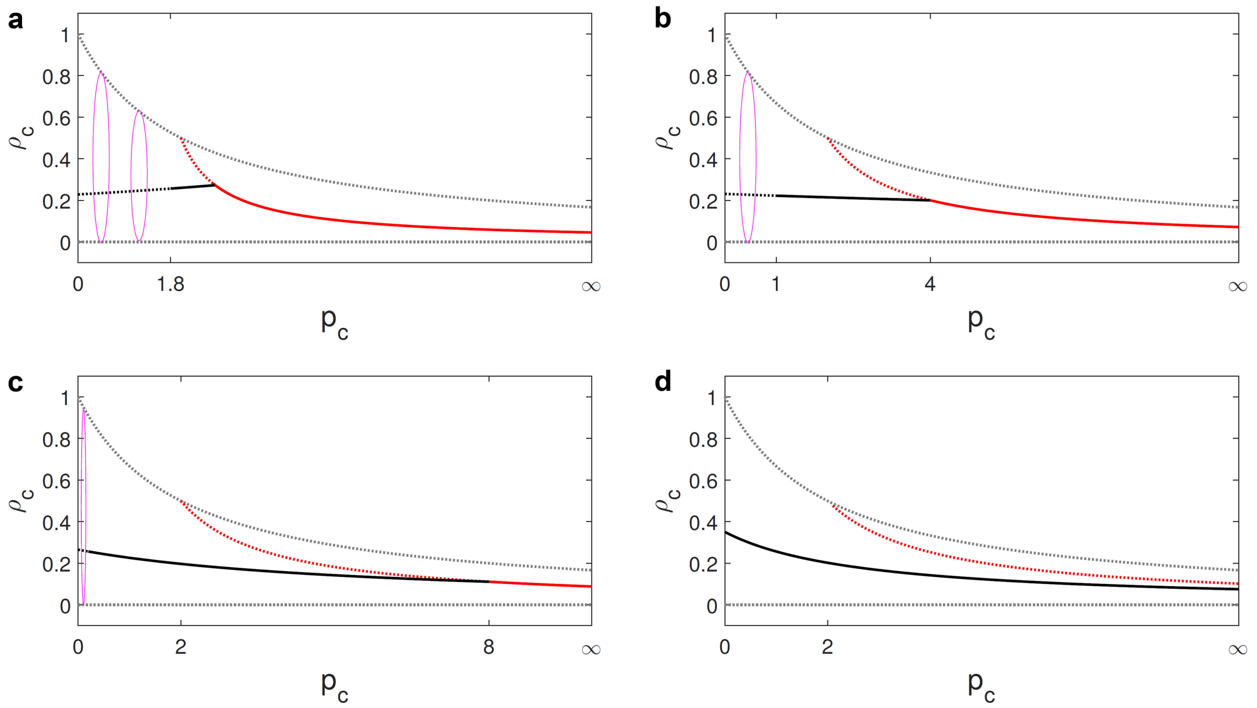

Supplementary Figure S2: Bifurcation diagrams of the RPS system (S4). For $p_a = p_b$, the values of parameter p_a from (**a**) to (**d**) are fixed at 0.5, 1.0, 1.5 and 2.1, respectively. Grey, red and black indicate three different states, $\mathbf{p}_1$, $\mathbf{p}_2$ and $\mathbf{p}_3$, respectively. Dotted and solid lines indicate unstable and stable fixed points, respectively. (**a**-**c**) As p_c is increased, after the stable heteroclinic cycles (ellipses) lose their stability, coexistence of all three species emerges, after which the coexistence state of two species is persistent. (**d**) For sufficiently large values of $p_a > 2$, the fixed point $\mathbf{p}_3$ is always stable, indicating persistent coexistence of three species.

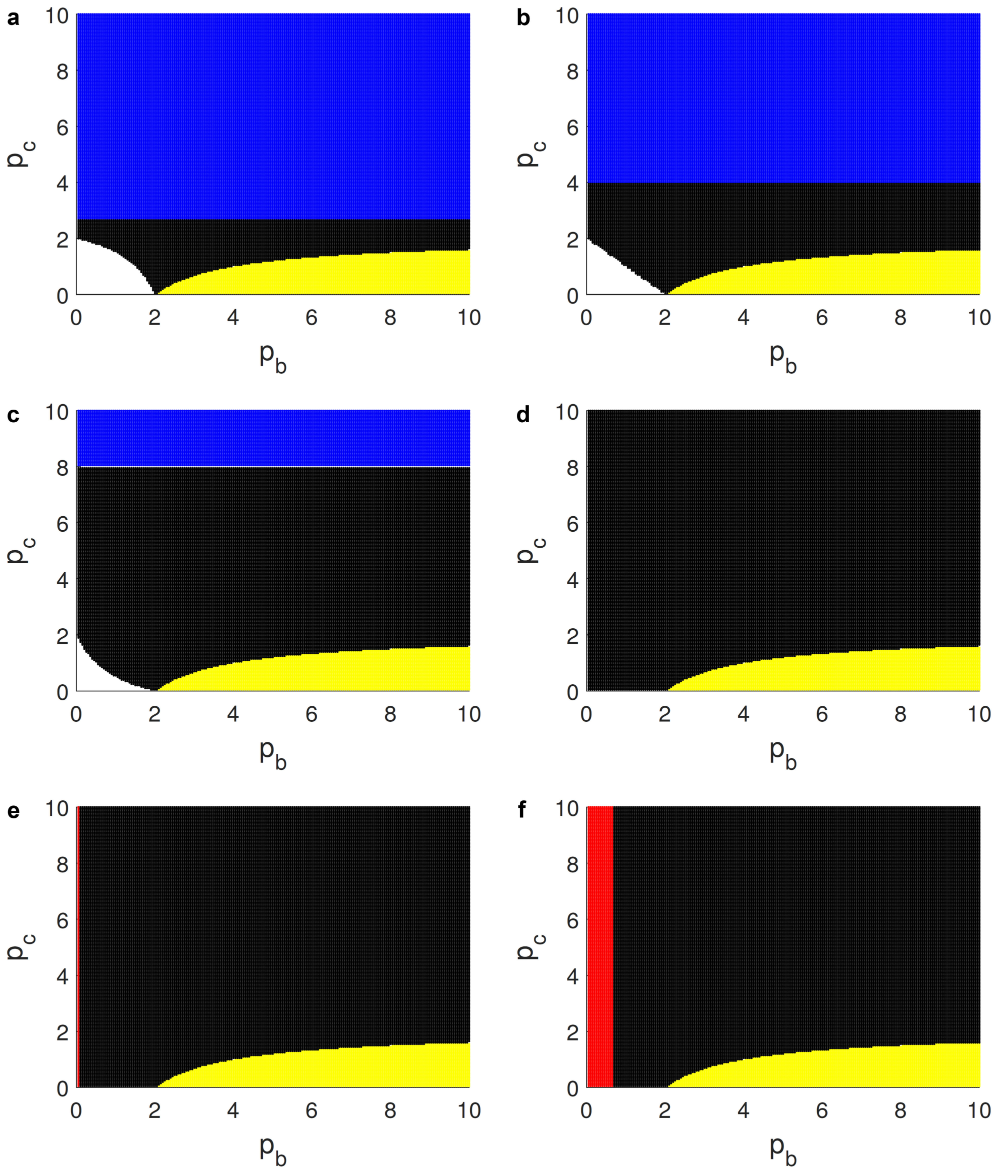

Supplementary Figure S3: Parameter regions of different attractors for fixed p_a. (**a**-**f**) For values of p_a fixed at 0.5, 1.0, 1.5, 2.0, 2.1 and 2.5, respectively, parameter regions of stable fixed points of type $\mathbf{p}_3$ and a stable heteroclinic cycle (black and blank regions, respectively). Red, blue and yellow areas indicate three different states of type $\mathbf{p}_2$ as stable attractors: AB, AC, and BC, respectively.

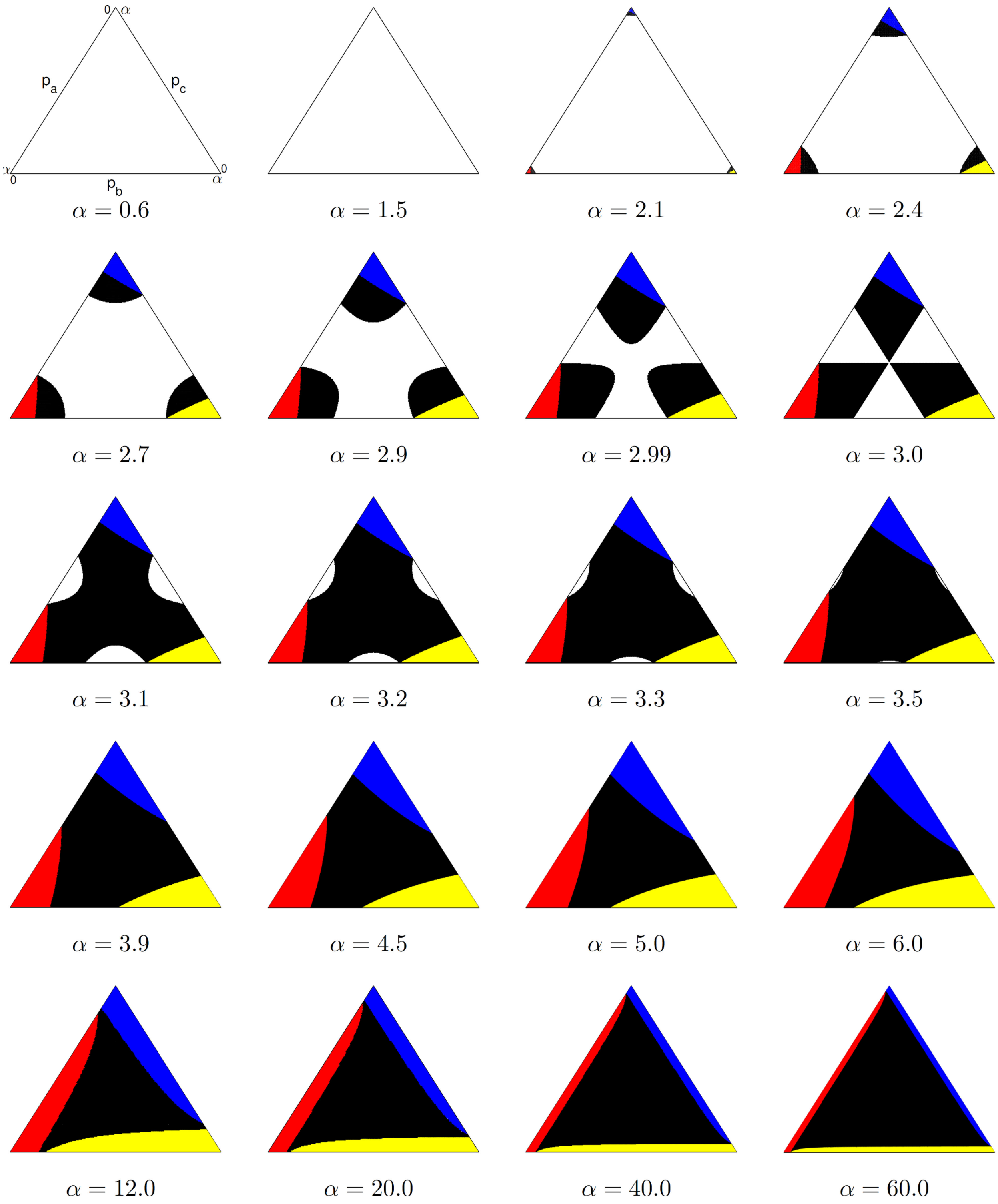

Supplementary Figure S4: Parameter basin versus total intraspecific competition rate $\alpha = p_a + p_b + p_c$ **on** 2**-simplex.** Each edge of the 2-simplex represents the parameters p_a, p_b and p_c, and its length is α. Black and blank regions indicate the parameter regions of the stable fixed point $\mathbf{p}_3$ and a stable heteroclinic cycle, respectively. Red, blue and yellow areas indicate three different states of type $\mathbf{p}_2$ as stable attractors: AB, AC, and BC, respectively.

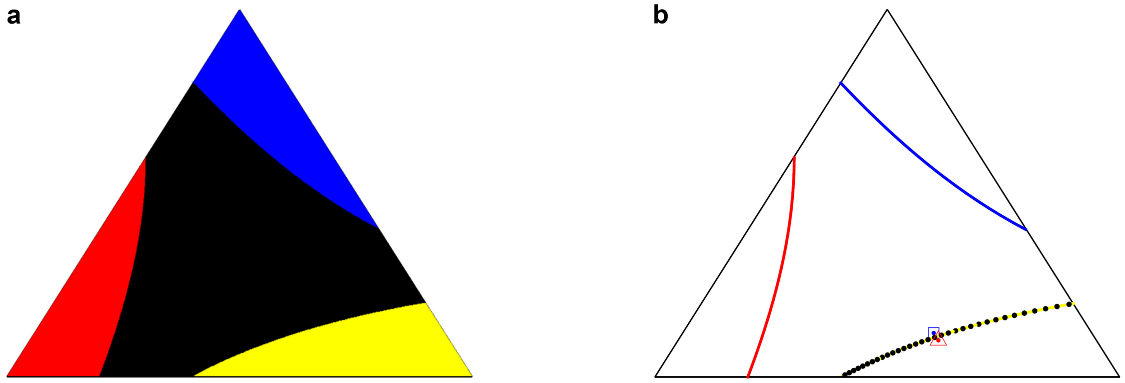

Supplementary Figure S5: Parameter basin and boundary curves for $p_a + p_b + p_c = 5$. Red, blue and yellow lines in (**b**) indicate the corresponding boundaries of the regions shown in (**a**). For instance, the yellow boundary is drawn from the equation $p_a + 4/(2 - p_c) + p_c = 5$.

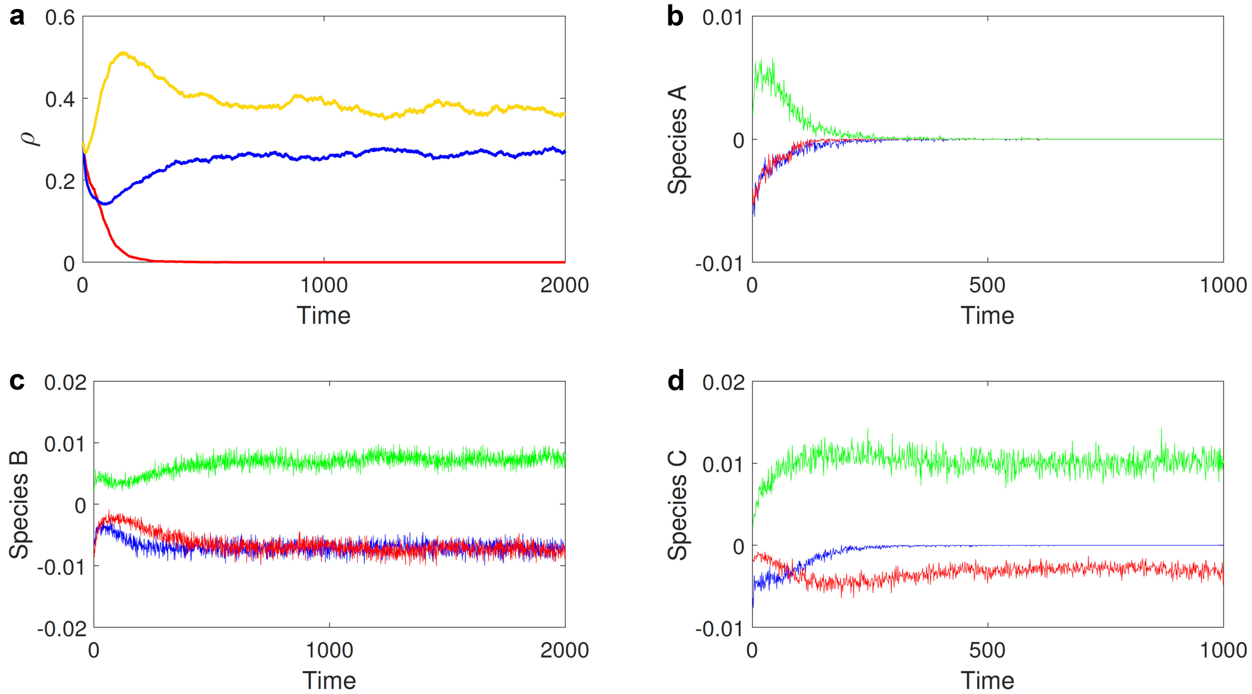

Supplementary Figure S6: Behaviors of densities from lattice simulation of RPS. For $p_a = 1.7$, $p_b = 2.8$, $p_c = 0.5$, and fixed mobility $M = 10^{-3}$, (**a**) densities of three species corresponding to extinction of species A and coexistence of species B and C. Red, blue and yellow lines indicate the densities of species A, B, and C, respectively. (**b-d**) Behaviors of species densities as a result of three types of interactions: interspecific competition (blue), intraspecific competition (red), and reproduction (green).

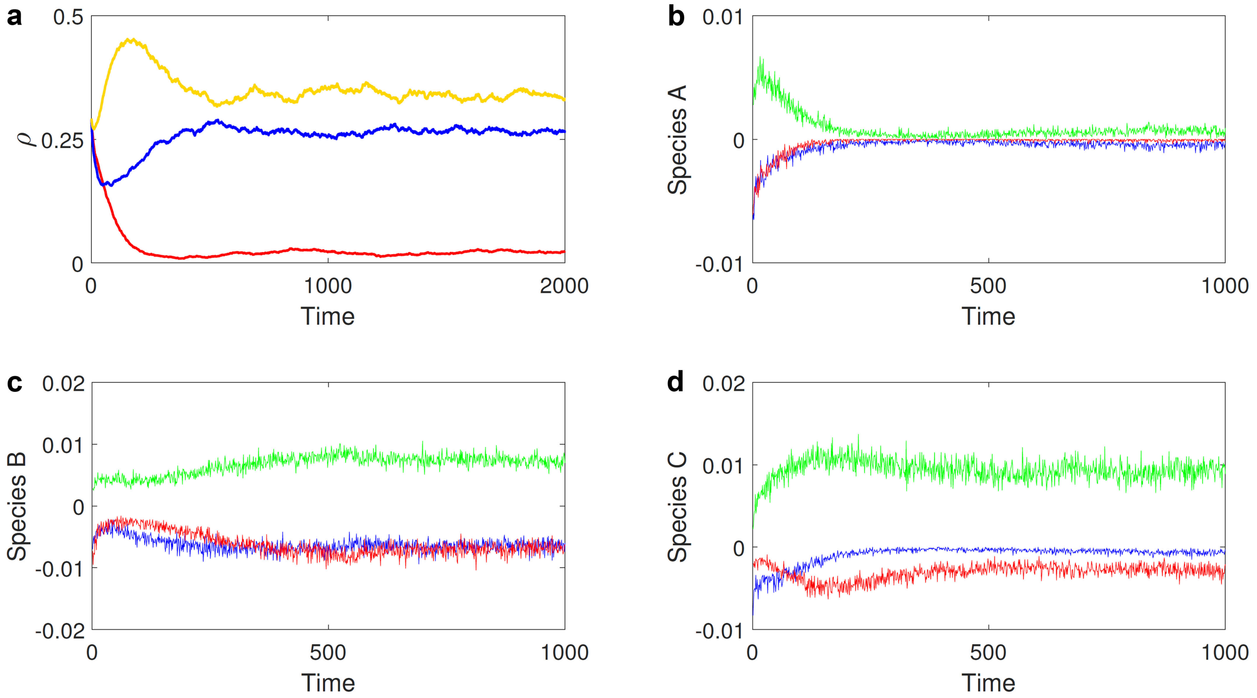

Supplementary Figure S7: Densities from lattice simulation of RPS system. For $p_a = 1.7$, $p_b = 2.7$, $p_c = 0.6$, and fixed mobility $M = 10^{-3}$, (**a**) densities of three species corresponding to extinction of species A and coexistence of species B and C. Red, blue and yellow lines indicate the densities of species A, B, and C, respectively. (**b-d**) Behaviors of species densities as a result of three types of interactions: interspecific competition (blue), intraspecific competition (red), and reproduction (green).

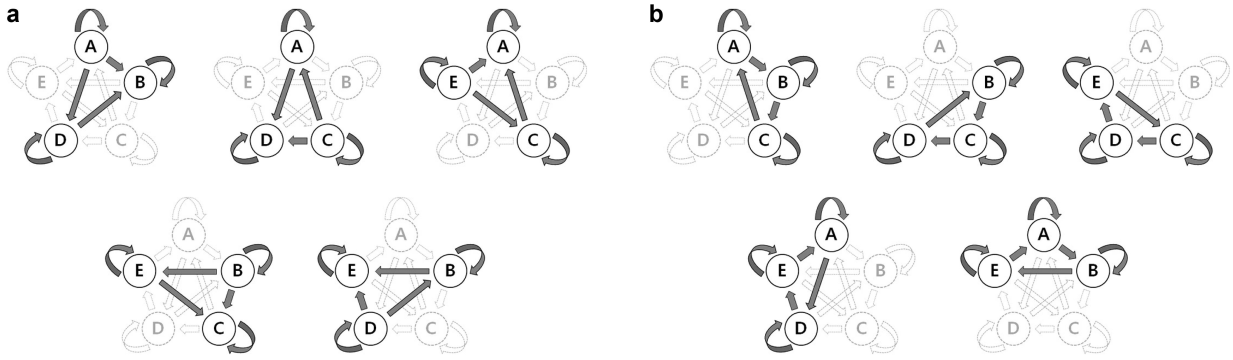

Supplementary Figure S8: Classification of fixed points of type $\mathbf{r}_3$ in RPSLS system (S23). There are ten fixed points of type $\mathbf{r}_3$. (**a**) Five possibly stable fixed points with a non-sub-cyclic structure, (**b**) other fixed points having a sub-cyclic structures, which are always unstable under the existence condition.

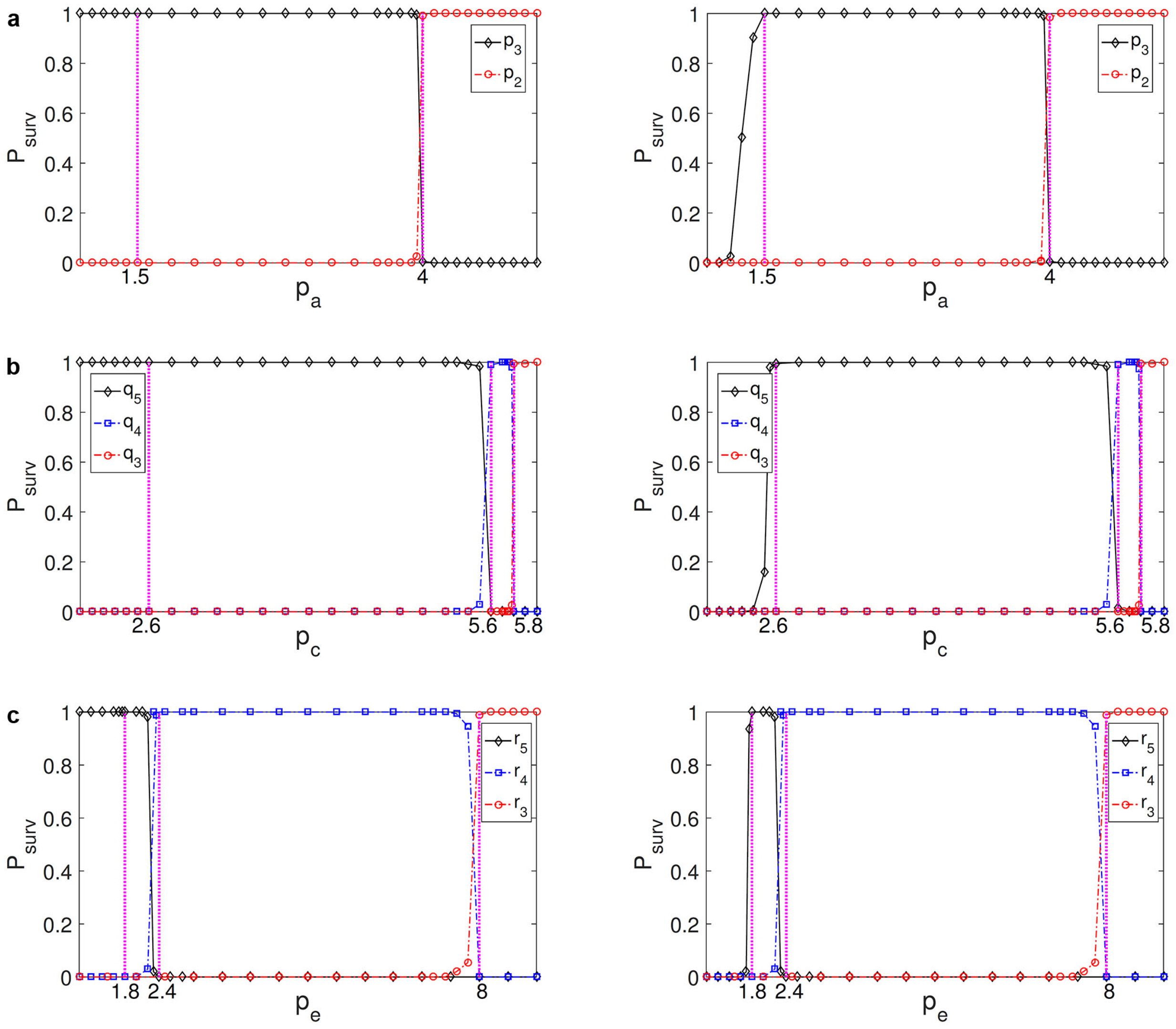

Supplementary Figure S9: Survival probability associated with each stable phase for different cyclic game systems. (a-c) For RPS, ERPS, and RPSLS systems, respectively, the survival probabilities for two different values of the mobility: $M = 10^{-5}$ (left) and $M = 10^{-3}$ (right). In each simulation, a square lattice of 500×500 sites is used and the survival probability is evaluated from 100 independent realizations. For each system, the thresholds of robust stable phases are consistent with those from the bifurcation analysis of the underlying ODE model.
